# *N*,*N*′-Bis(3β-acet­oxy-5α-cholest-6-yl­idene)hydrazine

**DOI:** 10.1107/S1600536811003254

**Published:** 2011-01-29

**Authors:** Zishan Tabassum, Othman Sulaiman, M. N. Mohamad Ibrahim, Ching Kheng Quah, Hoong-Kun Fun

**Affiliations:** a School of Industrial Technology, Universiti Sains Malaysia, 11800 USM, Penang, Malaysia; b School of Chemical Sciences, Universiti Sains Malaysia, 11800 USM, Penang, Malaysia; c X-ray Crystallography Unit, School of Physics, Universiti Sains Malaysia, 11800 USM, Penang, Malaysia

## Abstract

The asymmetric unit of the title compound, C_58_H_96_N_2_O_4_, contains two crystallographically independent mol­ecules. All cyclohexane rings are in chair conformations, while the furan ring is in an envelope conformation in one mol­ecule and a twist conformation in the other. Two acetaldehyde and one isobutane groups are disordered over two orientations with refined site occupancies of 0.940 (4):0.060 (4) and 0.791 (7):0.209 (7), respectively. In the crystal, mol­ecules are stacked along the *a* axis through van der Waals inter­actions.

## Related literature

For general background to the biological activity of steroids, see: Li *et al.* (1998 ▶); Fink *et al.* (1999 ▶). For the pharmacological activity of cholesterol derivatives, see: Khan *et al.* (2007 ▶); Shamsuzzaman *et al.* (2010 ▶). For the preparation of the title compound, see: Anagnostopoulos & Fieser (1954 ▶). For ring conformations, see: Cremer & Pople (1975 ▶). For the stability of the temperature controller used in the data collection, see: Cosier & Glazer (1986 ▶).
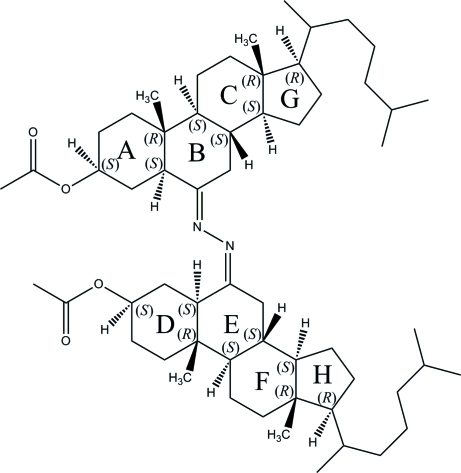


## Experimental

### 

#### Crystal data

C_58_H_96_N_2_O_4_
*M* *_r_* = 885.37
Triclinic, 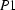

*a* = 9.5892 (4) Å
*b* = 16.1622 (6) Å
*c* = 19.5255 (7) Å
α = 65.832 (2)°
β = 89.562 (2)°
γ = 81.364 (2)°
*V* = 2724.70 (18) Å^3^
*Z* = 2
Cu *K*α radiation
μ = 0.50 mm^−1^
*T* = 100 K
0.55 × 0.47 × 0.31 mm

#### Data collection

Bruker APEX DUO CCD area-detector diffractometer
Absorption correction: multi-scan (*SADABS*; Bruker, 2009 ▶) *T*_min_ = 0.736, *T*_max_ = 0.860
109300 measured reflections
15864 independent reflections
15509 reflections with *I* > 2σ(*I*)
*R*_int_ = 0.037

#### Refinement

*R*[*F* ^2^ > 2σ(*F* ^2^)] = 0.057
*wR*(*F* ^2^) = 0.151
*S* = 1.19
15864 reflections
1266 parameters
258 restraints
H-atom parameters constrained
Δρ_max_ = 0.76 e Å^−3^
Δρ_min_ = −0.78 e Å^−3^
Absolute structure: Flack (1983 ▶), 6966 Friedel pairs
Flack parameter: −0.04 (17)

### 

Data collection: *APEX2* (Bruker, 2009 ▶); cell refinement: *SAINT* (Bruker, 2009 ▶); data reduction: *SAINT*; program(s) used to solve structure: *SHELXTL* (Sheldrick, 2008 ▶); program(s) used to refine structure: *SHELXTL*; molecular graphics: *SHELXTL*; software used to prepare material for publication: *SHELXTL* and *PLATON* (Spek, 2009 ▶).

## Supplementary Material

Crystal structure: contains datablocks global, I. DOI: 10.1107/S1600536811003254/rz2546sup1.cif

Structure factors: contains datablocks I. DOI: 10.1107/S1600536811003254/rz2546Isup2.hkl

Additional supplementary materials:  crystallographic information; 3D view; checkCIF report

## Figures and Tables

**Fig. 1.**
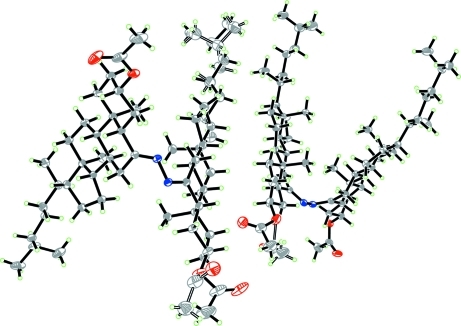
The asymmetric unit of the title compound showing 30% probability displacement ellipsoids for non-H atoms. Both major and minor components of disorder are shown.

**Fig. 2.**
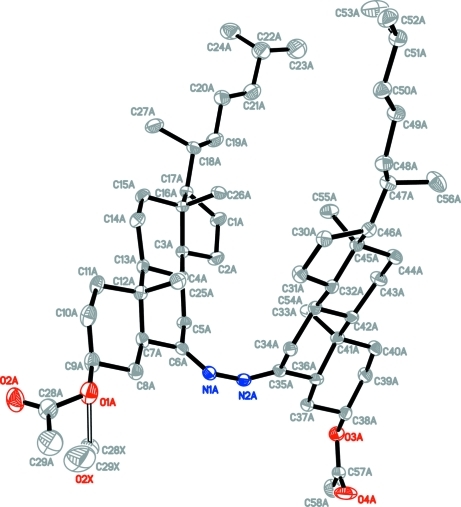
The molecular structure of the molecule A, showing 30% probability displacement ellipsoids and the atom-numbering scheme. All H atoms have been omitted for clarity. Both major and minor components of disorder are shown.

**Fig. 3.**
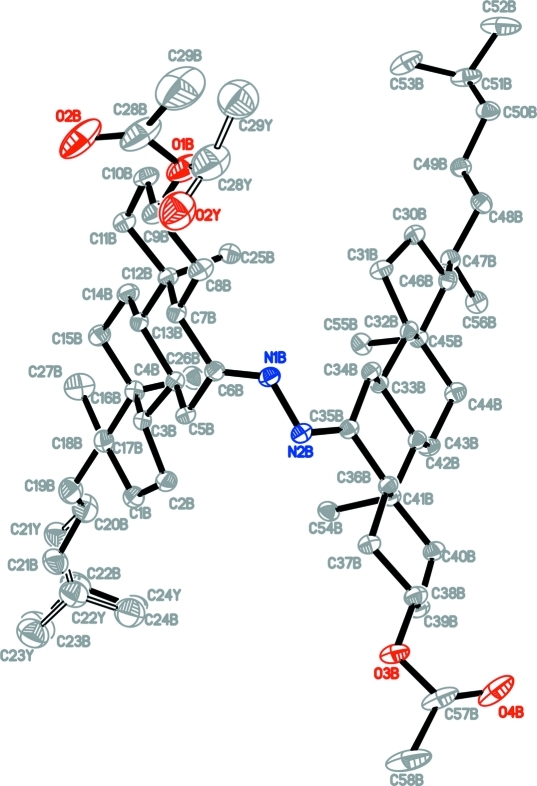
The molecular structure of the molecule B, showing 30% probability displacement ellipsoids and the atom-numbering scheme. All H atoms have been omitted for clarity. Both major and minor components of disorder are shown.

**Fig. 4.**
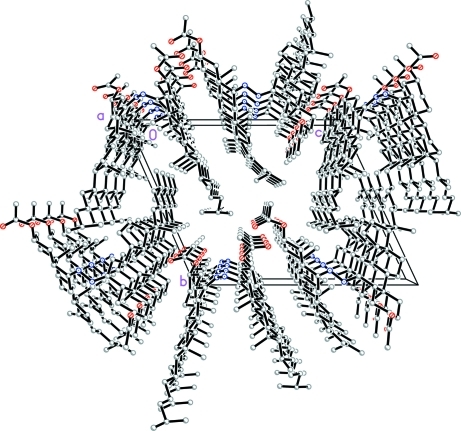
The crystal structure of the title compound, viewed along the a axis. All H atoms have been omitted for clarity. Only the major components of disorder are shown.

## supplementary crystallographic information

### Comment

Medicinal chemistry of steroids has become a matter of immense interest in recent past due to their biological applications (Li *et al.*, 1998; Fink *et al.*, 1999). The synthesis of cholesterol amine derivatives have also become of interest in recent years because their biocompatibility. Cholesterol intermediates and their derivatives are found to be pharmacologically active (Khan *et al.*, 2007; Shamsuzzaman *et al.*, 2010) which prompted us to synthesize a cholesterol derivative of hydrazine. Herein the synthesis and crystal structure of the title compound are reported.

The asymmetric unit (Fig. 1) contains two independent molecules, *A* and *B*. Each molecule is constructed from the fusion of six cyclohexane rings, A, B, C, D, E and F; and two furan rings, G and H. The absolute configuration at atoms C3, C4, C7, C9, C13, C32, C33, C36, C38, C42 is S and at atoms C12, C16, C17, C41, C45, C46 is R (see scheme, Fig. 2 and Fig. 3). The rings A/B, B/C, C/G, D/E, E/F and F/H are *trans*-fused. All cyclohexane rings (A/B/C/D/E/F) are in chair conformations. In molecule *A* (Fig. 2), the two furan rings, C1A-C3A/C16A/C17A and C30A-C32A/C45A/C46A are in envelope conformations, puckering parameters Q = 0.460 (3) Å; Θ = 288.6 (4)° (Cremer & Pople, 1975) with atom C16A at the flap; and Q = 0.466 (3) Å; Θ = 283.1 (3)° with atom C45A at the flap, respectively. In molecule *B* (Fig. 3), the two furan rings, C1B-C3B/C16B/C17B and C30B-C32B/C45B/C46B are in twist conformations, puckering parameters Q = 0.455 (3) Å; Θ = 273.4 (4)° with twisted on atoms C3B and C16B; and Q = 0.471 (3) Å; Θ = 272.7 (3)° with twisted on atoms C32B and C45B, respectively. The acetaldehyde group (O2/C28/C29) in both molecules and the isobutane (C21B-C24B) group in molecule *B* are disordered over two positions with refined site occupancies of 0.940 (4):0.060 (4) and 0.791 (7):0.209 (7), respectively.

In the crystal (Fig.4), molecules are stacked along the *a* axis and there is no significant hydrogen bond observed in this compound.

### Experimental

The title compound was prepared by reaction of 3β-acetoxy-5α-cholestan-6-one (0.0022 mol) (Anagnostopoulos & Fieser, 1954) with hydrazine hydrate (1.5 ml) in presence of glacial acetic acid (10 ml). Reaction progress was monitored by TLC. After refluxing the reaction mixture for 5 h, the solution was cooled and poured into ice cold water. The solution was extracted with chloroform; the organic layer was washed with water, sodium bicarbonate solution (5%) and passed through anhydrous sodium sulphate. The solution was concentrated under reduced pressure and the residue was crystallized from a chloroform–methanol (1:1) v/v solution (yield 70%). M.p. 226°C.

### Refinement

All H atoms were positioned geometrically and refined using a riding model with C–H = 0.96–0.98 Å and *U*_iso_(H) = 1.2 or 1.5 *U*_eq_(C). The highest residual electron density peak is located at 1.46 Å from H26D and the deepest hole is located at 1.43 Å from C3B. The acetaldehyde group (O2/C28/C29) in both molecules and the isobutane (C21B-C24B) group in molecule *B* are disordered over two positions with refined site occupancies of 0.940 (4):0.060 (4) and 0.791 (7):0.209 (7), respectively. All disordered atoms were subjected to rigid bond and similarity restraints. The same *U*_ij_ parameters were used for atom pair C29B/C29X.

### Crystal data

**Table:**

C_58_H_96_N_2_O_4_	*Z* = 2
*M**_r_* = 885.37	*F*(000) = 980
Triclinic, *P*1	*D*_x_ = 1.079 Mg m^−^^3^
Hall symbol: P 1	Cu *K*α radiation, λ = 1.54178 Å
*a* = 9.5892 (4) Å	Cell parameters from 9873 reflections
*b* = 16.1622 (6) Å	θ = 2.5–64.3°
*c* = 19.5255 (7) Å	µ = 0.50 mm^−^^1^
α = 65.832 (2)°	*T* = 100 K
β = 89.562 (2)°	Block, colourless
γ = 81.364 (2)°	0.55 × 0.47 × 0.31 mm
*V* = 2724.70 (18) Å^3^	

### Data collection

**Table:**

Bruker APEX DUO CCD area-detector diffractometer	15864 independent reflections
Radiation source: fine-focus sealed tube	15509 reflections with *I* > 2σ(*I*)
graphite	*R*_int_ = 0.037
φ and ω scans	θ_max_ = 65.0°, θ_min_ = 2.5°
Absorption correction: multi-scan (*SADABS*; Bruker, 2009)	*h* = −9→10
*T*_min_ = 0.736, *T*_max_ = 0.860	*k* = −18→18
109300 measured reflections	*l* = −22→22

### Refinement

**Table:**

Refinement on *F*^2^	Hydrogen site location: inferred from neighbouring sites
Least-squares matrix: full	H-atom parameters constrained
*R*[*F*^2^ > 2σ(*F*^2^)] = 0.057	*w* = 1/[σ^2^(*F*_o_^2^) + (0.1043*P*)^2^ + 0.2507*P*] where *P* = (*F*_o_^2^ + 2*F*_c_^2^)/3
*wR*(*F*^2^) = 0.151	(Δ/σ)_max_ = 0.002
*S* = 1.19	Δρ_max_ = 0.76 e Å^−^^3^
15864 reflections	Δρ_min_ = −0.78 e Å^−^^3^
1266 parameters	Extinction correction: *SHELXTL* (Sheldrick, 2008), Fc^*^=kFc[1+0.001xFc^2^λ^3^/sin(2θ)]^-1/4^
258 restraints	Extinction coefficient: 0.0332 (11)
Primary atom site location: structure-invariant direct methods	Absolute structure: Flack (1983), 6966 Friedel pairs
Secondary atom site location: difference Fourier map	Flack parameter: −0.04 (17)

### Special details

**Table:**

Experimental. The crystal was placed in the cold stream of an Oxford Cryosystems Cobra open-flow nitrogen cryostat (Cosier & Glazer, 1986) operating at 100.0 (1) K.
Geometry. All esds (except the esd in the dihedral angle between two l.s. planes) are estimated using the full covariance matrix. The cell esds are taken into account individually in the estimation of esds in distances, angles and torsion angles; correlations between esds in cell parameters are only used when they are defined by crystal symmetry. An approximate (isotropic) treatment of cell esds is used for estimating esds involving l.s. planes.
Refinement. Refinement of F^2^ against ALL reflections. The weighted R-factor wR and goodness of fit S are based on F^2^, conventional R-factors R are based on F, with F set to zero for negative F^2^. The threshold expression of F^2^ > 2sigma(F^2^) is used only for calculating R-factors(gt) etc. and is not relevant to the choice of reflections for refinement. R-factors based on F^2^ are statistically about twice as large as those based on F, and R- factors based on ALL data will be even larger.

### Fractional atomic coordinates and isotropic or equivalent isotropic displacement parameters (Å^2^)

**Table:**

	*x*	*y*	*z*	*U*_iso_*/*U*_eq_	Occ. (<1)
O1A	0.6010 (3)	0.62393 (14)	0.50502 (12)	0.0626 (6)	
O3A	0.6940 (2)	1.16240 (13)	0.72151 (10)	0.0455 (4)	
O4A	0.9115 (2)	1.08130 (19)	0.75787 (15)	0.0752 (7)	
N1A	0.5109 (2)	0.86582 (13)	0.60649 (10)	0.0348 (4)	
N2A	0.5282 (2)	0.92435 (13)	0.64225 (11)	0.0349 (4)	
C1A	−0.0887 (3)	1.1751 (2)	0.52255 (18)	0.0512 (7)	
H1AA	−0.0911	1.2400	0.4914	0.061*	
H1AB	−0.1256	1.1684	0.5706	0.061*	
C2A	0.0636 (3)	1.12323 (19)	0.53399 (17)	0.0468 (6)	
H2AA	0.1284	1.1647	0.5073	0.056*	
H2AB	0.0942	1.0934	0.5869	0.056*	
C3A	0.0561 (2)	1.05229 (16)	0.50167 (12)	0.0334 (5)	
H3AA	0.0672	1.0831	0.4475	0.040*	
C4A	0.1643 (2)	0.96519 (15)	0.53132 (12)	0.0317 (5)	
H4AA	0.1498	0.9307	0.5847	0.038*	
C5A	0.3172 (3)	0.98540 (15)	0.52466 (13)	0.0345 (5)	
H5AA	0.3317	1.0239	0.4728	0.041*	
H5AB	0.3331	1.0184	0.5550	0.041*	
C6A	0.4203 (3)	0.89720 (16)	0.55079 (12)	0.0338 (5)	
C7A	0.3997 (3)	0.83939 (15)	0.50880 (12)	0.0357 (5)	
H7AA	0.4046	0.8788	0.4554	0.043*	
C8A	0.5158 (3)	0.75660 (17)	0.52705 (14)	0.0429 (6)	
H8AA	0.6069	0.7771	0.5174	0.051*	
H8AB	0.5168	0.7159	0.5799	0.051*	
C9A	0.4914 (3)	0.70470 (18)	0.47941 (15)	0.0487 (7)	
H9AA	0.5025	0.7435	0.4265	0.058*	
C10A	0.3480 (4)	0.67648 (18)	0.48732 (16)	0.0536 (7)	
H10A	0.3412	0.6316	0.5382	0.064*	
H10B	0.3349	0.6480	0.4532	0.064*	
C11A	0.2315 (3)	0.76026 (17)	0.46959 (14)	0.0443 (6)	
H11A	0.1404	0.7396	0.4774	0.053*	
H11B	0.2324	0.8015	0.4170	0.053*	
C12A	0.2481 (3)	0.81389 (15)	0.51816 (12)	0.0353 (5)	
C13A	0.1402 (3)	0.90488 (15)	0.48919 (12)	0.0337 (5)	
H13A	0.1602	0.9397	0.4367	0.040*	
C14A	−0.0141 (3)	0.89071 (17)	0.48784 (14)	0.0419 (6)	
H14A	−0.0339	0.8481	0.5375	0.050*	
H14B	−0.0266	0.8629	0.4532	0.050*	
C15A	−0.1215 (3)	0.97977 (17)	0.46445 (14)	0.0397 (5)	
H15A	−0.1141	1.0180	0.4116	0.048*	
H15B	−0.2163	0.9647	0.4710	0.048*	
C16A	−0.0967 (2)	1.03375 (16)	0.51100 (12)	0.0340 (5)	
C17A	−0.1782 (3)	1.13257 (17)	0.48342 (13)	0.0369 (5)	
H17A	−0.1658	1.1630	0.4294	0.044*	
C18A	−0.3384 (3)	1.14694 (19)	0.49380 (13)	0.0429 (6)	
H18A	−0.3567	1.0961	0.5403	0.051*	
C19A	−0.3961 (3)	1.2347 (2)	0.50048 (19)	0.0582 (7)	
H19A	−0.3759	1.2856	0.4554	0.070*	
H19B	−0.3456	1.2364	0.5426	0.070*	
C20A	−0.5534 (3)	1.2500 (2)	0.51133 (17)	0.0562 (7)	
H20A	−0.6043	1.2698	0.4631	0.067*	
H20B	−0.5799	1.1918	0.5445	0.067*	
C21A	−0.5992 (4)	1.3177 (3)	0.5424 (2)	0.0686 (9)	
H21A	−0.5750	1.3762	0.5081	0.082*	
H21B	−0.5450	1.2990	0.5895	0.082*	
C22A	−0.7566 (4)	1.3332 (3)	0.5570 (2)	0.0726 (9)	
H22A	−0.7810	1.2722	0.5866	0.087*	
C23A	−0.7826 (6)	1.3861 (4)	0.6051 (3)	0.1024 (15)	
H23A	−0.8792	1.3884	0.6184	0.154*	
H23B	−0.7635	1.4474	0.5777	0.154*	
H23C	−0.7214	1.3562	0.6500	0.154*	
C24A	−0.8505 (4)	1.3735 (3)	0.4883 (2)	0.0770 (10)	
H24A	−0.9470	1.3795	0.5014	0.115*	
H24B	−0.8368	1.3344	0.4620	0.115*	
H24C	−0.8290	1.4330	0.4565	0.115*	
C25A	0.2282 (3)	0.75307 (17)	0.60077 (13)	0.0414 (5)	
H25A	0.1369	0.7344	0.6050	0.062*	
H25B	0.2350	0.7871	0.6307	0.062*	
H25C	0.3002	0.6997	0.6183	0.062*	
C26A	−0.1238 (3)	0.9770 (2)	0.59372 (14)	0.0466 (6)	
H26A	−0.0598	0.9198	0.6126	0.070*	
H26B	−0.2193	0.9651	0.5972	0.070*	
H26C	−0.1092	1.0107	0.6230	0.070*	
C27A	−0.4188 (4)	1.1406 (3)	0.4285 (2)	0.0672 (9)	
H27A	−0.5172	1.1414	0.4381	0.101*	
H27B	−0.3812	1.0845	0.4243	0.101*	
H27C	−0.4081	1.1919	0.3824	0.101*	
C30A	0.1409 (3)	0.61277 (16)	0.87933 (13)	0.0378 (5)	
H30A	0.1895	0.5503	0.9064	0.045*	
H30B	0.0555	0.6110	0.8541	0.045*	
C31A	0.2364 (3)	0.67036 (16)	0.82194 (14)	0.0398 (5)	
H31A	0.3249	0.6328	0.8212	0.048*	
H31B	0.1902	0.6984	0.7719	0.048*	
C32A	0.2615 (2)	0.74348 (15)	0.84887 (12)	0.0320 (5)	
H32A	0.3355	0.7137	0.8896	0.038*	
C33A	0.3107 (3)	0.83052 (15)	0.79395 (12)	0.0324 (5)	
H33A	0.2366	0.8642	0.7537	0.039*	
C34A	0.4479 (3)	0.80871 (15)	0.75851 (13)	0.0349 (5)	
H34A	0.5181	0.7678	0.7980	0.042*	
H34B	0.4291	0.7776	0.7271	0.042*	
C35A	0.5048 (2)	0.89506 (15)	0.71188 (12)	0.0334 (5)	
C36A	0.5283 (3)	0.95204 (16)	0.75477 (13)	0.0350 (5)	
H36A	0.5893	0.9111	0.7995	0.042*	
C37A	0.6048 (3)	1.03295 (17)	0.71249 (13)	0.0365 (5)	
H37A	0.6941	1.0111	0.6971	0.044*	
H37B	0.5480	1.0759	0.6676	0.044*	
C38A	0.6315 (3)	1.08154 (17)	0.76234 (13)	0.0381 (5)	
H38A	0.6943	1.0394	0.8059	0.046*	
C39A	0.4938 (3)	1.11442 (18)	0.78907 (14)	0.0423 (6)	
H39A	0.4354	1.1606	0.7462	0.051*	
H39B	0.5136	1.1425	0.8226	0.051*	
C40A	0.4137 (3)	1.03434 (17)	0.83028 (14)	0.0395 (5)	
H40A	0.3242	1.0581	0.8442	0.047*	
H40B	0.4680	0.9923	0.8763	0.047*	
C41A	0.3849 (2)	0.98064 (15)	0.78374 (13)	0.0339 (5)	
C42A	0.3328 (2)	0.89091 (15)	0.83510 (13)	0.0334 (5)	
H42A	0.4092	0.8558	0.8737	0.040*	
C43A	0.2025 (3)	0.90670 (17)	0.87743 (16)	0.0428 (6)	
H43A	0.2250	0.9390	0.9072	0.051*	
H43B	0.1258	0.9454	0.8411	0.051*	
C44A	0.1527 (3)	0.81670 (17)	0.92951 (15)	0.0412 (6)	
H44A	0.2246	0.7809	0.9696	0.049*	
H44B	0.0672	0.8309	0.9521	0.049*	
C45A	0.1238 (2)	0.75990 (15)	0.88642 (12)	0.0322 (5)	
C46A	0.1039 (3)	0.65876 (15)	0.93479 (12)	0.0342 (5)	
H46A	0.1787	0.6327	0.9752	0.041*	
C47A	−0.0374 (3)	0.64195 (17)	0.97238 (14)	0.0394 (5)	
H47A	−0.1132	0.6841	0.9360	0.047*	
C48A	−0.0666 (3)	0.54393 (17)	0.99405 (14)	0.0412 (5)	
H48A	−0.0514	0.5285	0.9512	0.049*	
H48B	0.0008	0.5017	1.0345	0.049*	
C49A	−0.2143 (3)	0.53015 (17)	1.01885 (15)	0.0430 (6)	
H49A	−0.2815	0.5801	0.9833	0.052*	
H49B	−0.2231	0.5330	1.0674	0.052*	
C50A	−0.2525 (3)	0.43924 (18)	1.02499 (15)	0.0452 (6)	
H50A	−0.1804	0.3899	1.0572	0.054*	
H50B	−0.2503	0.4388	0.9755	0.054*	
C51A	−0.3965 (3)	0.4190 (2)	1.05572 (15)	0.0480 (6)	
H51A	−0.4000	0.4234	1.1043	0.058*	
C52A	−0.4142 (4)	0.3223 (2)	1.06891 (17)	0.0576 (7)	
H52A	−0.3385	0.2794	1.1025	0.086*	
H52B	−0.4125	0.3165	1.0219	0.086*	
H52C	−0.5029	0.3096	1.0907	0.086*	
C53A	−0.5173 (4)	0.4875 (3)	1.0046 (2)	0.0756 (10)	
H53A	−0.5082	0.5482	0.9992	0.113*	
H53B	−0.6054	0.4723	1.0260	0.113*	
H53C	−0.5149	0.4854	0.9561	0.113*	
C54A	0.2773 (3)	1.04003 (17)	0.71781 (15)	0.0408 (5)	
H54A	0.1879	1.0530	0.7369	0.061*	
H54B	0.2663	1.0076	0.6871	0.061*	
H54C	0.3103	1.0966	0.6882	0.061*	
C55A	−0.0016 (3)	0.81067 (16)	0.82875 (15)	0.0433 (6)	
H55A	0.0203	0.8684	0.7934	0.065*	
H55B	−0.0838	0.8213	0.8542	0.065*	
H55C	−0.0197	0.7740	0.8026	0.065*	
C56A	−0.0433 (4)	0.6640 (2)	1.04120 (19)	0.0629 (9)	
H56A	−0.1339	0.6563	1.0620	0.094*	
H56B	−0.0291	0.7264	1.0268	0.094*	
H56C	0.0295	0.6233	1.0781	0.094*	
C57A	0.8347 (3)	1.1534 (2)	0.72545 (15)	0.0486 (6)	
C58A	0.8836 (4)	1.2440 (3)	0.68515 (19)	0.0676 (9)	
H58A	0.9790	1.2341	0.6714	0.101*	
H58B	0.8798	1.2757	0.7175	0.101*	
H58C	0.8235	1.2804	0.6406	0.101*	
O1B	0.8082 (2)	−0.20779 (17)	0.28307 (16)	0.0701 (7)	
O3B	−0.2866 (2)	−0.15170 (13)	0.06624 (11)	0.0486 (4)	
O4B	−0.3658 (3)	−0.16249 (17)	−0.03740 (16)	0.0794 (8)	
N1B	0.3059 (2)	−0.09523 (12)	0.17311 (10)	0.0315 (4)	
N2B	0.1741 (2)	−0.08582 (12)	0.13787 (10)	0.0311 (4)	
C1B	−0.1279 (3)	0.2235 (2)	0.25264 (16)	0.0472 (6)	
H1BA	−0.1863	0.1977	0.2946	0.057*	
H1BB	−0.1880	0.2692	0.2103	0.057*	
C2B	−0.0511 (3)	0.1476 (2)	0.23152 (15)	0.0445 (6)	
H2BA	−0.0885	0.0908	0.2568	0.053*	
H2BB	−0.0596	0.1658	0.1776	0.053*	
C3B	0.1013 (3)	0.13700 (16)	0.25826 (12)	0.0340 (5)	
H3BA	0.1052	0.1039	0.3131	0.041*	
C4B	0.2170 (3)	0.08400 (16)	0.23112 (12)	0.0332 (5)	
H4BA	0.2225	0.1186	0.1769	0.040*	
C5B	0.1847 (3)	−0.01204 (16)	0.24504 (12)	0.0339 (5)	
H5BA	0.1675	−0.0449	0.2976	0.041*	
H5BB	0.1004	−0.0060	0.2151	0.041*	
C6B	0.3076 (2)	−0.06498 (14)	0.22419 (11)	0.0308 (5)	
C7B	0.4452 (3)	−0.07534 (16)	0.26557 (12)	0.0341 (5)	
H7BA	0.4281	−0.1028	0.3192	0.041*	
C8B	0.5649 (3)	−0.14081 (18)	0.25352 (15)	0.0409 (5)	
H8BA	0.5362	−0.2003	0.2670	0.049*	
H8BB	0.5860	−0.1170	0.2009	0.049*	
C9B	0.6961 (3)	−0.15132 (19)	0.30167 (16)	0.0469 (6)	
H9BA	0.6777	−0.1826	0.3549	0.056*	
C10B	0.7409 (3)	−0.06084 (19)	0.28785 (16)	0.0451 (6)	
H10C	0.7713	−0.0334	0.2370	0.054*	
H10D	0.8202	−0.0708	0.3224	0.054*	
C11B	0.6187 (3)	0.00477 (18)	0.29869 (14)	0.0400 (5)	
H11C	0.6492	0.0633	0.2874	0.048*	
H11D	0.5956	−0.0202	0.3510	0.048*	
C12B	0.4851 (3)	0.02096 (16)	0.24895 (12)	0.0343 (5)	
C13B	0.3599 (2)	0.07757 (15)	0.27043 (11)	0.0320 (5)	
H13B	0.3505	0.0439	0.3245	0.038*	
C14B	0.3862 (3)	0.17332 (17)	0.25901 (14)	0.0403 (5)	
H14C	0.4009	0.2079	0.2063	0.048*	
H14D	0.4721	0.1671	0.2878	0.048*	
C15B	0.2650 (3)	0.22773 (17)	0.28261 (14)	0.0402 (5)	
H15C	0.2573	0.1974	0.3365	0.048*	
H15D	0.2858	0.2888	0.2710	0.048*	
C16B	0.1241 (3)	0.23559 (16)	0.24230 (13)	0.0375 (5)	
C17B	−0.0122 (3)	0.26796 (18)	0.27476 (14)	0.0423 (6)	
H17B	0.0022	0.2386	0.3297	0.051*	
C18B	−0.0618 (3)	0.3707 (2)	0.25245 (16)	0.0523 (7)	
H18B	−0.0869	0.3994	0.1983	0.063*	
C19B	−0.1955 (4)	0.3850 (2)	0.29275 (19)	0.0623 (8)	
H19C	−0.2502	0.3370	0.2981	0.075*	
H19D	−0.1666	0.3765	0.3430	0.075*	
C20B	−0.2905 (4)	0.4757 (3)	0.2563 (2)	0.0735 (10)	
H20C	−0.3131	0.4880	0.2043	0.088*	0.791 (7)
H20D	−0.2412	0.5238	0.2565	0.088*	0.791 (7)
H20E	−0.3408	0.4727	0.2152	0.088*	0.209 (7)
H20F	−0.2269	0.5188	0.2335	0.088*	0.209 (7)
C21B	−0.4303 (6)	0.4780 (4)	0.2973 (3)	0.0685 (13)	0.791 (7)
H21C	−0.4698	0.4231	0.3053	0.082*	0.791 (7)
H21D	−0.4081	0.4762	0.3463	0.082*	0.791 (7)
C22B	−0.5417 (6)	0.5617 (4)	0.2552 (3)	0.0833 (14)	0.791 (7)
H22B	−0.4957	0.6162	0.2409	0.100*	0.791 (7)
C23B	−0.6593 (9)	0.5698 (7)	0.3069 (5)	0.121 (2)	0.791 (7)
H23D	−0.7201	0.6279	0.2825	0.181*	0.791 (7)
H23E	−0.6182	0.5656	0.3531	0.181*	0.791 (7)
H23F	−0.7133	0.5210	0.3174	0.181*	0.791 (7)
C24B	−0.6017 (7)	0.5619 (5)	0.1842 (4)	0.0908 (17)	0.791 (7)
H24D	−0.5261	0.5473	0.1565	0.136*	0.791 (7)
H24E	−0.6538	0.6216	0.1541	0.136*	0.791 (7)
H24F	−0.6635	0.5169	0.1967	0.136*	0.791 (7)
C21Y	−0.385 (2)	0.5201 (17)	0.2836 (13)	0.073 (3)	0.209 (7)
H21E	−0.3991	0.5860	0.2536	0.088*	0.209 (7)
H21F	−0.3611	0.5071	0.3355	0.088*	0.209 (7)
C22Y	−0.516 (3)	0.4776 (16)	0.2761 (13)	0.082 (3)	0.209 (7)
H22C	−0.4905	0.4105	0.2971	0.098*	0.209 (7)
C23Y	−0.635 (3)	0.505 (2)	0.3260 (16)	0.104 (5)	0.209 (7)
H23G	−0.6615	0.4498	0.3636	0.155*	0.209 (7)
H23H	−0.7169	0.5417	0.2940	0.155*	0.209 (7)
H23I	−0.5978	0.5387	0.3499	0.155*	0.209 (7)
C24Y	−0.588 (3)	0.511 (2)	0.1984 (16)	0.096 (5)	0.209 (7)
H24G	−0.5277	0.5438	0.1611	0.144*	0.209 (7)
H24H	−0.6756	0.5506	0.1949	0.144*	0.209 (7)
H24I	−0.6075	0.4590	0.1899	0.144*	0.209 (7)
C25B	0.5150 (3)	0.07071 (17)	0.16590 (13)	0.0379 (5)	
H25D	0.5649	0.1203	0.1598	0.057*	
H25E	0.4274	0.0946	0.1360	0.057*	
H25F	0.5716	0.0283	0.1499	0.057*	
C26B	0.1253 (3)	0.29551 (19)	0.15720 (14)	0.0475 (6)	
H26D	0.1994	0.2681	0.1361	0.071*	
H26E	0.1412	0.3559	0.1495	0.071*	
H26F	0.0360	0.2999	0.1330	0.071*	
C27B	0.0516 (4)	0.4195 (2)	0.2675 (2)	0.0695 (9)	
H27D	0.1292	0.4177	0.2365	0.104*	
H27E	0.0846	0.3891	0.3195	0.104*	
H27F	0.0121	0.4823	0.2559	0.104*	
C30B	0.4664 (3)	0.27825 (15)	−0.10755 (13)	0.0353 (5)	
H30C	0.5395	0.2561	−0.1334	0.042*	
H30D	0.5038	0.3181	−0.0889	0.042*	
C31B	0.4176 (3)	0.19689 (15)	−0.04211 (13)	0.0363 (5)	
H31C	0.4815	0.1401	−0.0318	0.044*	
H31D	0.4119	0.2077	0.0033	0.044*	
C32B	0.2728 (2)	0.19374 (14)	−0.07058 (11)	0.0291 (4)	
H32B	0.2895	0.1693	−0.1088	0.035*	
C33B	0.1768 (2)	0.13454 (14)	−0.01583 (11)	0.0282 (4)	
H33B	0.1540	0.1583	0.0224	0.034*	
C34B	0.2524 (2)	0.03429 (14)	0.02353 (11)	0.0293 (4)	
H34C	0.2908	0.0138	−0.0140	0.035*	
H34D	0.3304	0.0313	0.0562	0.035*	
C35B	0.1531 (2)	−0.02890 (14)	0.06929 (11)	0.0283 (4)	
C36B	0.0186 (2)	−0.02208 (14)	0.02679 (12)	0.0292 (4)	
H36B	0.0474	−0.0358	−0.0162	0.035*	
C37B	−0.0742 (3)	−0.09340 (15)	0.07140 (13)	0.0341 (5)	
H37C	−0.0193	−0.1546	0.0898	0.041*	
H37D	−0.1080	−0.0823	0.1143	0.041*	
C38B	−0.1979 (3)	−0.08626 (16)	0.02082 (14)	0.0373 (5)	
H38B	−0.1635	−0.1023	−0.0203	0.045*	
C39B	−0.2839 (3)	0.00909 (17)	−0.01137 (14)	0.0388 (5)	
H39C	−0.3254	0.0226	0.0291	0.047*	
H39D	−0.3601	0.0117	−0.0449	0.047*	
C40B	−0.1921 (3)	0.08079 (16)	−0.05427 (13)	0.0352 (5)	
H40C	−0.2492	0.1413	−0.0720	0.042*	
H40D	−0.1600	0.0710	−0.0980	0.042*	
C41B	−0.0625 (2)	0.07858 (14)	−0.00713 (12)	0.0295 (4)	
C42B	0.0386 (2)	0.14065 (14)	−0.05869 (11)	0.0288 (4)	
H42B	0.0659	0.1157	−0.0958	0.035*	
C43B	−0.0286 (3)	0.24143 (15)	−0.10391 (13)	0.0365 (5)	
H43C	−0.1105	0.2433	−0.1336	0.044*	
H43D	−0.0610	0.2682	−0.0692	0.044*	
C44B	0.0724 (3)	0.29949 (15)	−0.15619 (13)	0.0367 (5)	
H44C	0.0254	0.3627	−0.1809	0.044*	
H44D	0.0962	0.2774	−0.1948	0.044*	
C45B	0.2088 (2)	0.29616 (14)	−0.11371 (12)	0.0308 (5)	
C46B	0.3339 (3)	0.33129 (14)	−0.16192 (12)	0.0323 (5)	
H46B	0.3364	0.3092	−0.2018	0.039*	
C47B	0.3407 (3)	0.43498 (15)	−0.19976 (13)	0.0356 (5)	
H47B	0.3498	0.4559	−0.1597	0.043*	
C48B	0.4736 (3)	0.45176 (16)	−0.24527 (14)	0.0404 (5)	
H48C	0.5457	0.3978	−0.2227	0.048*	
H48D	0.4504	0.4593	−0.2960	0.048*	
C49B	0.5345 (3)	0.53560 (17)	−0.24954 (14)	0.0402 (5)	
H49C	0.4650	0.5901	−0.2755	0.048*	
H49D	0.5513	0.5303	−0.1989	0.048*	
C50B	0.6707 (3)	0.54787 (19)	−0.28941 (15)	0.0467 (6)	
H50C	0.6535	0.5548	−0.3405	0.056*	
H50D	0.7398	0.4929	−0.2642	0.056*	
C51B	0.7322 (3)	0.6312 (2)	−0.29161 (19)	0.0610 (8)	
H51B	0.6577	0.6850	−0.3126	0.073*	
C52B	0.8545 (4)	0.6493 (3)	−0.3433 (3)	0.0965 (16)	
H52D	0.8963	0.6985	−0.3409	0.145*	
H52E	0.8201	0.6660	−0.3940	0.145*	
H52F	0.9242	0.5948	−0.3275	0.145*	
C53B	0.7772 (4)	0.6199 (3)	−0.2132 (2)	0.0806 (11)	
H53D	0.8165	0.6728	−0.2169	0.121*	
H53E	0.8470	0.5658	−0.1902	0.121*	
H53F	0.6965	0.6142	−0.1831	0.121*	
C54B	−0.1093 (3)	0.10896 (16)	0.05558 (13)	0.0362 (5)	
H54D	−0.1586	0.1710	0.0337	0.054*	
H54E	−0.0277	0.1056	0.0854	0.054*	
H54F	−0.1708	0.0692	0.0869	0.054*	
C55B	0.1763 (3)	0.34464 (15)	−0.06056 (13)	0.0382 (5)	
H55D	0.1182	0.3110	−0.0220	0.057*	
H55E	0.1272	0.4059	−0.0887	0.057*	
H55F	0.2631	0.3472	−0.0379	0.057*	
C56B	0.2090 (3)	0.49238 (16)	−0.24913 (14)	0.0429 (6)	
H56D	0.1294	0.4888	−0.2185	0.064*	
H56E	0.1915	0.4693	−0.2859	0.064*	
H56F	0.2229	0.5552	−0.2742	0.064*	
C57B	−0.3688 (4)	−0.1821 (2)	0.0289 (2)	0.0642 (9)	
C58B	−0.4649 (5)	−0.2412 (3)	0.0833 (3)	0.0953 (15)	
H58D	−0.5190	−0.2665	0.0577	0.143*	
H58E	−0.5277	−0.2043	0.1021	0.143*	
H58F	−0.4087	−0.2903	0.1245	0.143*	
O2A	0.6156 (3)	0.62990 (19)	0.38859 (15)	0.0745 (8)	0.940 (4)
C28A	0.6525 (4)	0.5945 (2)	0.4535 (2)	0.0644 (9)	0.940 (4)
C29A	0.7654 (6)	0.5119 (3)	0.4893 (3)	0.0970 (16)	0.940 (4)
H29A	0.7810	0.4805	0.4569	0.146*	0.940 (4)
H29B	0.7359	0.4712	0.5367	0.146*	0.940 (4)
H29C	0.8514	0.5312	0.4973	0.146*	0.940 (4)
O2B	0.8981 (4)	−0.2704 (2)	0.4043 (2)	0.1012 (12)	0.940 (4)
C28B	0.9021 (5)	−0.2644 (3)	0.3407 (3)	0.0903 (14)	0.940 (4)
C29B	1.0096 (7)	−0.3174 (5)	0.3112 (5)	0.137 (2)	0.940 (4)
H29D	1.0590	−0.3701	0.3525	0.205*	0.940 (4)
H29E	1.0758	−0.2788	0.2835	0.205*	0.940 (4)
H29F	0.9628	−0.3372	0.2786	0.205*	0.940 (4)
O2Y	0.767 (6)	−0.338 (3)	0.325 (3)	0.088 (8)	0.060 (4)
C28Y	0.844 (9)	−0.279 (6)	0.274 (5)	0.093 (5)	0.060 (4)
C29Y	0.982 (10)	−0.313 (7)	0.252 (6)	0.104 (8)	0.060 (4)
H29G	1.0351	−0.2637	0.2307	0.156*	0.060 (4)
H29H	0.9666	−0.3377	0.2162	0.156*	0.060 (4)
H29I	1.0341	−0.3609	0.2961	0.156*	0.060 (4)
O2X	0.794 (3)	0.709 (3)	0.4639 (16)	0.056 (10)	0.060 (4)
C28X	0.771 (7)	0.626 (6)	0.503 (3)	0.08 (2)	0.060 (4)
C29X	0.816 (12)	0.524 (8)	0.528 (8)	0.137 (2)	0.060 (4)
H29J	0.8828	0.5132	0.4943	0.205*	0.060 (4)
H29K	0.7347	0.4964	0.5269	0.205*	0.060 (4)
H29L	0.8589	0.4981	0.5778	0.205*	0.060 (4)

### Atomic displacement parameters (Å^2^)

**Table:**

	*U*^11^	*U*^22^	*U*^33^	*U*^12^	*U*^13^	*U*^23^
O1A	0.0735 (16)	0.0539 (12)	0.0594 (12)	0.0200 (10)	−0.0139 (10)	−0.0323 (10)
O3A	0.0417 (12)	0.0523 (10)	0.0496 (10)	−0.0184 (8)	0.0057 (8)	−0.0246 (8)
O4A	0.0310 (13)	0.0931 (18)	0.0855 (16)	−0.0121 (12)	0.0098 (10)	−0.0204 (14)
N1A	0.0333 (11)	0.0386 (10)	0.0362 (10)	−0.0074 (8)	0.0067 (8)	−0.0188 (8)
N2A	0.0267 (11)	0.0402 (10)	0.0439 (11)	−0.0051 (7)	0.0034 (8)	−0.0236 (8)
C1A	0.0353 (16)	0.0619 (16)	0.0702 (17)	0.0026 (12)	−0.0079 (12)	−0.0445 (14)
C2A	0.0340 (15)	0.0508 (14)	0.0656 (16)	0.0003 (11)	−0.0063 (11)	−0.0363 (13)
C3A	0.0280 (13)	0.0398 (12)	0.0360 (11)	−0.0074 (9)	0.0034 (9)	−0.0187 (9)
C4A	0.0291 (13)	0.0389 (11)	0.0309 (10)	−0.0086 (9)	0.0045 (8)	−0.0174 (9)
C5A	0.0293 (13)	0.0354 (11)	0.0418 (12)	−0.0078 (9)	0.0041 (9)	−0.0182 (9)
C6A	0.0324 (13)	0.0375 (11)	0.0336 (11)	−0.0092 (9)	0.0097 (9)	−0.0158 (9)
C7A	0.0416 (15)	0.0356 (11)	0.0278 (10)	−0.0031 (10)	0.0056 (9)	−0.0121 (9)
C8A	0.0456 (16)	0.0415 (12)	0.0403 (12)	0.0039 (11)	−0.0004 (10)	−0.0194 (10)
C9A	0.062 (2)	0.0405 (13)	0.0426 (13)	0.0065 (12)	−0.0017 (12)	−0.0209 (11)
C10A	0.075 (2)	0.0378 (13)	0.0518 (15)	0.0005 (12)	−0.0093 (13)	−0.0257 (12)
C11A	0.0573 (18)	0.0371 (12)	0.0414 (12)	−0.0056 (11)	−0.0055 (11)	−0.0196 (10)
C12A	0.0389 (14)	0.0341 (11)	0.0335 (11)	−0.0059 (9)	0.0010 (9)	−0.0147 (9)
C13A	0.0381 (14)	0.0367 (11)	0.0282 (10)	−0.0104 (9)	0.0029 (9)	−0.0139 (9)
C14A	0.0442 (16)	0.0401 (12)	0.0447 (13)	−0.0094 (11)	−0.0041 (10)	−0.0198 (10)
C15A	0.0359 (15)	0.0446 (13)	0.0425 (12)	−0.0113 (10)	−0.0023 (10)	−0.0201 (10)
C16A	0.0258 (13)	0.0448 (12)	0.0326 (11)	−0.0074 (9)	0.0022 (8)	−0.0167 (9)
C17A	0.0294 (14)	0.0477 (13)	0.0384 (11)	−0.0064 (10)	0.0036 (9)	−0.0226 (10)
C18A	0.0290 (14)	0.0586 (15)	0.0369 (12)	−0.0026 (10)	0.0034 (9)	−0.0170 (11)
C19A	0.0399 (18)	0.0711 (19)	0.0714 (19)	−0.0062 (14)	−0.0041 (13)	−0.0380 (16)
C20A	0.0422 (18)	0.0675 (18)	0.0546 (16)	0.0027 (13)	0.0003 (12)	−0.0248 (14)
C21A	0.055 (2)	0.081 (2)	0.075 (2)	−0.0071 (17)	−0.0053 (16)	−0.0387 (18)
C22A	0.056 (2)	0.080 (2)	0.090 (2)	0.0052 (17)	0.0018 (17)	−0.048 (2)
C23A	0.078 (3)	0.135 (4)	0.121 (4)	−0.007 (3)	0.008 (3)	−0.084 (3)
C24A	0.049 (2)	0.086 (2)	0.098 (3)	0.0040 (17)	0.0026 (18)	−0.045 (2)
C25A	0.0435 (15)	0.0383 (12)	0.0377 (12)	−0.0087 (10)	0.0017 (10)	−0.0101 (10)
C26A	0.0289 (15)	0.0667 (16)	0.0383 (12)	−0.0054 (11)	0.0042 (10)	−0.0166 (12)
C27A	0.0366 (18)	0.093 (2)	0.094 (2)	−0.0069 (15)	−0.0031 (15)	−0.061 (2)
C30A	0.0400 (15)	0.0333 (11)	0.0443 (12)	−0.0077 (9)	0.0029 (10)	−0.0196 (10)
C31A	0.0482 (16)	0.0354 (11)	0.0443 (12)	−0.0121 (10)	0.0084 (10)	−0.0234 (10)
C32A	0.0310 (13)	0.0351 (11)	0.0339 (11)	−0.0038 (9)	−0.0002 (9)	−0.0186 (9)
C33A	0.0308 (13)	0.0346 (11)	0.0378 (11)	−0.0058 (9)	0.0017 (9)	−0.0207 (9)
C34A	0.0340 (14)	0.0376 (11)	0.0402 (12)	−0.0052 (9)	0.0040 (9)	−0.0235 (10)
C35A	0.0268 (13)	0.0386 (11)	0.0385 (12)	−0.0037 (9)	0.0036 (9)	−0.0203 (9)
C36A	0.0265 (13)	0.0439 (12)	0.0404 (12)	−0.0079 (9)	0.0030 (9)	−0.0225 (10)
C37A	0.0307 (13)	0.0455 (12)	0.0388 (11)	−0.0097 (10)	0.0062 (9)	−0.0218 (10)
C38A	0.0403 (15)	0.0427 (12)	0.0340 (11)	−0.0093 (10)	−0.0007 (9)	−0.0176 (10)
C39A	0.0474 (16)	0.0447 (13)	0.0451 (13)	−0.0165 (11)	0.0084 (11)	−0.0258 (11)
C40A	0.0441 (15)	0.0422 (12)	0.0431 (12)	−0.0152 (10)	0.0117 (10)	−0.0257 (10)
C41A	0.0290 (13)	0.0379 (11)	0.0428 (12)	−0.0106 (9)	0.0068 (9)	−0.0230 (10)
C42A	0.0284 (13)	0.0395 (12)	0.0409 (12)	−0.0083 (9)	0.0043 (9)	−0.0242 (10)
C43A	0.0407 (15)	0.0460 (13)	0.0624 (15)	−0.0186 (11)	0.0209 (12)	−0.0396 (12)
C44A	0.0419 (15)	0.0464 (13)	0.0519 (14)	−0.0176 (11)	0.0164 (11)	−0.0336 (11)
C45A	0.0303 (13)	0.0319 (10)	0.0398 (11)	−0.0074 (9)	0.0025 (9)	−0.0195 (9)
C46A	0.0344 (13)	0.0324 (11)	0.0360 (11)	−0.0044 (9)	−0.0017 (9)	−0.0145 (9)
C47A	0.0376 (15)	0.0369 (11)	0.0439 (12)	−0.0068 (10)	0.0071 (10)	−0.0167 (10)
C48A	0.0402 (15)	0.0376 (12)	0.0467 (13)	−0.0091 (10)	0.0029 (10)	−0.0172 (10)
C49A	0.0417 (16)	0.0406 (12)	0.0467 (13)	−0.0101 (10)	0.0058 (11)	−0.0169 (10)
C50A	0.0446 (16)	0.0440 (13)	0.0457 (13)	−0.0123 (11)	0.0001 (11)	−0.0155 (11)
C51A	0.0458 (17)	0.0541 (15)	0.0441 (13)	−0.0165 (12)	0.0049 (11)	−0.0175 (11)
C52A	0.057 (2)	0.0659 (18)	0.0519 (15)	−0.0309 (15)	−0.0006 (13)	−0.0188 (13)
C53A	0.042 (2)	0.073 (2)	0.097 (3)	−0.0162 (15)	−0.0038 (17)	−0.0169 (19)
C54A	0.0341 (14)	0.0373 (12)	0.0521 (13)	−0.0094 (9)	0.0069 (10)	−0.0185 (10)
C55A	0.0346 (15)	0.0358 (12)	0.0530 (14)	−0.0076 (10)	−0.0008 (11)	−0.0112 (10)
C56A	0.081 (2)	0.0611 (17)	0.0707 (19)	−0.0401 (16)	0.0393 (17)	−0.0423 (16)
C57A	0.0350 (17)	0.0783 (19)	0.0448 (13)	−0.0183 (14)	0.0080 (11)	−0.0348 (13)
C58A	0.071 (2)	0.091 (2)	0.0636 (18)	−0.049 (2)	0.0232 (16)	−0.0436 (17)
O1B	0.0419 (14)	0.0757 (14)	0.1021 (17)	0.0129 (10)	−0.0225 (11)	−0.0532 (13)
O3B	0.0352 (11)	0.0481 (10)	0.0631 (11)	−0.0179 (8)	−0.0021 (8)	−0.0198 (8)
O4B	0.083 (2)	0.0648 (14)	0.0890 (18)	−0.0286 (12)	−0.0387 (14)	−0.0240 (13)
N1B	0.0278 (11)	0.0311 (9)	0.0321 (9)	−0.0061 (7)	−0.0022 (7)	−0.0092 (7)
N2B	0.0275 (11)	0.0318 (9)	0.0358 (9)	−0.0038 (7)	−0.0027 (7)	−0.0159 (8)
C1B	0.0378 (16)	0.0643 (16)	0.0467 (13)	−0.0062 (12)	0.0048 (10)	−0.0309 (12)
C2B	0.0332 (15)	0.0627 (16)	0.0484 (13)	−0.0077 (11)	0.0046 (10)	−0.0339 (12)
C3B	0.0332 (13)	0.0428 (12)	0.0286 (10)	−0.0100 (9)	0.0025 (8)	−0.0162 (9)
C4B	0.0301 (13)	0.0412 (12)	0.0292 (10)	−0.0074 (9)	0.0007 (8)	−0.0148 (9)
C5B	0.0288 (13)	0.0441 (12)	0.0319 (10)	−0.0130 (9)	0.0017 (8)	−0.0162 (9)
C6B	0.0319 (13)	0.0300 (10)	0.0273 (10)	−0.0096 (9)	0.0011 (8)	−0.0070 (8)
C7B	0.0338 (14)	0.0395 (12)	0.0280 (10)	−0.0084 (9)	−0.0004 (9)	−0.0121 (9)
C8B	0.0334 (15)	0.0433 (12)	0.0465 (13)	−0.0036 (10)	−0.0062 (10)	−0.0198 (10)
C9B	0.0346 (15)	0.0501 (14)	0.0545 (15)	−0.0006 (11)	−0.0104 (11)	−0.0219 (12)
C10B	0.0287 (15)	0.0581 (15)	0.0520 (14)	−0.0074 (11)	−0.0042 (10)	−0.0261 (12)
C11B	0.0337 (14)	0.0487 (13)	0.0410 (12)	−0.0119 (10)	−0.0005 (10)	−0.0202 (10)
C12B	0.0288 (13)	0.0413 (12)	0.0327 (11)	−0.0103 (9)	−0.0006 (9)	−0.0137 (9)
C13B	0.0312 (13)	0.0384 (11)	0.0261 (10)	−0.0108 (9)	0.0012 (8)	−0.0113 (8)
C14B	0.0363 (15)	0.0434 (13)	0.0413 (12)	−0.0122 (10)	−0.0007 (10)	−0.0155 (10)
C15B	0.0407 (15)	0.0386 (12)	0.0440 (12)	−0.0101 (10)	0.0000 (10)	−0.0184 (10)
C16B	0.0385 (15)	0.0395 (12)	0.0350 (12)	−0.0067 (10)	0.0018 (9)	−0.0158 (10)
C17B	0.0434 (16)	0.0487 (14)	0.0375 (12)	−0.0063 (11)	0.0038 (10)	−0.0210 (10)
C18B	0.0543 (19)	0.0532 (15)	0.0535 (15)	−0.0020 (13)	0.0032 (13)	−0.0285 (13)
C19B	0.062 (2)	0.0704 (19)	0.0652 (18)	−0.0025 (15)	0.0073 (15)	−0.0414 (16)
C20B	0.069 (3)	0.081 (2)	0.074 (2)	0.0084 (18)	0.0033 (18)	−0.0422 (19)
C21B	0.066 (3)	0.079 (3)	0.072 (2)	−0.011 (2)	0.018 (2)	−0.043 (2)
C22B	0.066 (3)	0.087 (3)	0.101 (3)	−0.010 (2)	0.022 (2)	−0.044 (2)
C23B	0.083 (4)	0.137 (5)	0.138 (5)	0.008 (4)	0.026 (4)	−0.061 (4)
C24B	0.061 (3)	0.096 (4)	0.108 (4)	−0.031 (3)	0.004 (3)	−0.029 (3)
C21Y	0.074 (5)	0.076 (5)	0.082 (5)	−0.014 (4)	0.016 (4)	−0.043 (4)
C22Y	0.073 (5)	0.087 (5)	0.087 (5)	−0.013 (4)	0.014 (4)	−0.038 (4)
C23Y	0.083 (7)	0.115 (8)	0.104 (7)	−0.011 (7)	0.019 (6)	−0.039 (6)
C24Y	0.086 (8)	0.102 (9)	0.093 (7)	−0.017 (7)	0.008 (6)	−0.034 (7)
C25B	0.0350 (14)	0.0437 (12)	0.0374 (12)	−0.0132 (10)	0.0065 (9)	−0.0167 (10)
C26B	0.0455 (17)	0.0489 (14)	0.0401 (13)	−0.0011 (11)	0.0035 (11)	−0.0127 (11)
C27B	0.067 (2)	0.0508 (17)	0.098 (3)	−0.0050 (15)	0.0037 (18)	−0.0395 (17)
C30B	0.0339 (14)	0.0350 (11)	0.0387 (11)	−0.0080 (9)	0.0003 (9)	−0.0161 (9)
C31B	0.0314 (14)	0.0325 (11)	0.0415 (12)	−0.0072 (9)	−0.0049 (9)	−0.0110 (9)
C32B	0.0302 (13)	0.0294 (10)	0.0303 (10)	−0.0030 (8)	−0.0009 (8)	−0.0154 (8)
C33B	0.0288 (12)	0.0289 (10)	0.0305 (10)	−0.0051 (8)	−0.0004 (8)	−0.0157 (8)
C34B	0.0268 (12)	0.0310 (10)	0.0312 (10)	−0.0035 (8)	−0.0017 (8)	−0.0144 (8)
C35B	0.0285 (12)	0.0272 (10)	0.0322 (10)	−0.0032 (8)	−0.0002 (8)	−0.0157 (8)
C36B	0.0261 (12)	0.0325 (10)	0.0328 (10)	−0.0044 (8)	0.0004 (8)	−0.0176 (8)
C37B	0.0304 (13)	0.0343 (11)	0.0394 (11)	−0.0087 (9)	0.0005 (9)	−0.0160 (9)
C38B	0.0323 (14)	0.0393 (12)	0.0449 (12)	−0.0111 (9)	0.0012 (10)	−0.0202 (10)
C39B	0.0267 (13)	0.0463 (13)	0.0467 (13)	−0.0074 (10)	−0.0021 (9)	−0.0219 (10)
C40B	0.0268 (13)	0.0382 (11)	0.0419 (12)	−0.0030 (9)	−0.0023 (9)	−0.0187 (10)
C41B	0.0237 (12)	0.0333 (11)	0.0351 (11)	−0.0035 (8)	−0.0007 (8)	−0.0179 (9)
C42B	0.0258 (12)	0.0319 (10)	0.0310 (10)	−0.0021 (8)	−0.0003 (8)	−0.0161 (8)
C43B	0.0292 (13)	0.0344 (11)	0.0414 (12)	−0.0019 (9)	−0.0039 (9)	−0.0123 (9)
C44B	0.0359 (14)	0.0306 (11)	0.0368 (11)	−0.0032 (9)	−0.0049 (9)	−0.0078 (9)
C45B	0.0303 (13)	0.0298 (10)	0.0327 (10)	−0.0043 (8)	−0.0002 (8)	−0.0134 (9)
C46B	0.0371 (14)	0.0297 (11)	0.0323 (10)	−0.0054 (9)	0.0006 (9)	−0.0150 (9)
C47B	0.0403 (14)	0.0310 (11)	0.0361 (11)	−0.0082 (9)	0.0043 (9)	−0.0138 (9)
C48B	0.0431 (15)	0.0362 (11)	0.0399 (12)	−0.0074 (10)	0.0078 (10)	−0.0135 (9)
C49B	0.0341 (14)	0.0444 (13)	0.0412 (12)	−0.0075 (10)	0.0008 (10)	−0.0163 (10)
C50B	0.0343 (15)	0.0481 (14)	0.0447 (13)	−0.0035 (11)	−0.0001 (10)	−0.0073 (11)
C51B	0.0331 (17)	0.0498 (15)	0.0766 (19)	−0.0073 (12)	−0.0103 (13)	−0.0021 (14)
C52B	0.035 (2)	0.087 (3)	0.117 (3)	−0.0186 (17)	0.0048 (19)	0.011 (2)
C53B	0.060 (3)	0.078 (2)	0.098 (3)	−0.0217 (18)	−0.0283 (19)	−0.027 (2)
C54B	0.0323 (13)	0.0382 (11)	0.0433 (12)	−0.0060 (9)	0.0084 (9)	−0.0221 (10)
C55B	0.0461 (15)	0.0295 (10)	0.0425 (12)	−0.0077 (9)	0.0075 (10)	−0.0177 (9)
C56B	0.0493 (17)	0.0314 (11)	0.0426 (12)	−0.0062 (10)	0.0025 (11)	−0.0100 (9)
C57B	0.045 (2)	0.0477 (15)	0.094 (3)	−0.0179 (13)	−0.0230 (16)	−0.0204 (16)
C58B	0.062 (3)	0.079 (2)	0.132 (4)	−0.045 (2)	−0.014 (2)	−0.018 (2)
O2A	0.088 (2)	0.0755 (16)	0.0719 (16)	0.0088 (13)	0.0014 (13)	−0.0493 (13)
C28A	0.063 (2)	0.0605 (19)	0.082 (2)	0.0079 (15)	−0.0056 (17)	−0.0479 (18)
C29A	0.090 (3)	0.081 (3)	0.124 (4)	0.036 (2)	−0.020 (3)	−0.063 (3)
O2B	0.085 (2)	0.087 (2)	0.126 (3)	0.0257 (16)	−0.067 (2)	−0.0514 (19)
C28B	0.063 (3)	0.086 (3)	0.133 (3)	0.017 (2)	−0.043 (2)	−0.065 (3)
C29B	0.093 (4)	0.137 (4)	0.184 (5)	0.051 (3)	−0.041 (4)	−0.093 (4)
O2Y	0.091 (10)	0.087 (11)	0.099 (11)	−0.007 (8)	−0.004 (8)	−0.053 (8)
C28Y	0.080 (7)	0.093 (7)	0.114 (7)	0.000 (6)	−0.016 (6)	−0.055 (6)
C29Y	0.094 (9)	0.103 (11)	0.117 (10)	0.000 (8)	0.001 (8)	−0.053 (8)
O2X	0.035 (19)	0.08 (2)	0.034 (14)	0.016 (16)	0.004 (12)	−0.021 (15)
C28X	0.06 (4)	0.17 (7)	0.05 (3)	−0.07 (4)	0.04 (3)	−0.05 (4)
C29X	0.093 (4)	0.137 (4)	0.184 (5)	0.051 (3)	−0.041 (4)	−0.093 (4)

### Geometric parameters (Å, °)

**Table:**

O1A—C28A	1.340 (4)	C3B—H3BA	0.9800
O1A—C9A	1.456 (3)	C4B—C5B	1.542 (3)
O1A—C28X	1.64 (6)	C4B—C13B	1.545 (3)
O3A—C57A	1.334 (4)	C4B—H4BA	0.9800
O3A—C38A	1.442 (3)	C5B—C6B	1.504 (3)
O4A—C57A	1.202 (4)	C5B—H5BA	0.9700
N1A—C6A	1.275 (3)	C5B—H5BB	0.9700
N1A—N2A	1.414 (3)	C6B—C7B	1.504 (3)
N2A—C35A	1.274 (3)	C7B—C8B	1.526 (3)
C1A—C2A	1.539 (4)	C7B—C12B	1.561 (3)
C1A—C17A	1.552 (4)	C7B—H7BA	0.9800
C1A—H1AA	0.9700	C8B—C9B	1.523 (4)
C1A—H1AB	0.9700	C8B—H8BA	0.9700
C2A—C3A	1.528 (3)	C8B—H8BB	0.9700
C2A—H2AA	0.9700	C9B—C10B	1.503 (4)
C2A—H2AB	0.9700	C9B—H9BA	0.9800
C3A—C4A	1.515 (3)	C10B—C11B	1.533 (4)
C3A—C16A	1.534 (3)	C10B—H10C	0.9700
C3A—H3AA	0.9800	C10B—H10D	0.9700
C4A—C5A	1.543 (3)	C11B—C12B	1.537 (3)
C4A—C13A	1.552 (3)	C11B—H11C	0.9700
C4A—H4AA	0.9800	C11B—H11D	0.9700
C5A—C6A	1.503 (3)	C12B—C25B	1.534 (3)
C5A—H5AA	0.9700	C12B—C13B	1.559 (3)
C5A—H5AB	0.9700	C13B—C14B	1.530 (3)
C6A—C7A	1.507 (3)	C13B—H13B	0.9800
C7A—C8A	1.525 (3)	C14B—C15B	1.534 (4)
C7A—C12A	1.559 (4)	C14B—H14C	0.9700
C7A—H7AA	0.9800	C14B—H14D	0.9700
C8A—C9A	1.525 (4)	C15B—C16B	1.531 (4)
C8A—H8AA	0.9700	C15B—H15C	0.9700
C8A—H8AB	0.9700	C15B—H15D	0.9700
C9A—C10A	1.500 (5)	C16B—C26B	1.544 (3)
C9A—H9AA	0.9800	C16B—C17B	1.561 (3)
C10A—C11A	1.539 (4)	C17B—C18B	1.532 (4)
C10A—H10A	0.9700	C17B—H17B	0.9800
C10A—H10B	0.9700	C18B—C27B	1.531 (5)
C11A—C12A	1.547 (3)	C18B—C19B	1.536 (4)
C11A—H11A	0.9700	C18B—H18B	0.9800
C11A—H11B	0.9700	C19B—C20B	1.497 (5)
C12A—C25A	1.536 (3)	C19B—H19C	0.9700
C12A—C13A	1.557 (3)	C19B—H19D	0.9700
C13A—C14A	1.532 (4)	C20B—C21Y	1.32 (2)
C13A—H13A	0.9800	C20B—C21B	1.560 (6)
C14A—C15A	1.538 (4)	C20B—H20C	0.9700
C14A—H14A	0.9700	C20B—H20D	0.9700
C14A—H14B	0.9700	C20B—H20E	0.9606
C15A—C16A	1.536 (3)	C20B—H20F	0.9618
C15A—H15A	0.9700	C21B—C22B	1.523 (8)
C15A—H15B	0.9700	C21B—H21C	0.9700
C16A—C26A	1.539 (3)	C21B—H21D	0.9700
C16A—C17A	1.543 (3)	C22B—C24B	1.505 (9)
C17A—C18A	1.543 (4)	C22B—C23B	1.534 (8)
C17A—H17A	0.9800	C22B—H22B	0.9800
C18A—C19A	1.499 (4)	C23B—H23D	0.9600
C18A—C27A	1.542 (4)	C23B—H23E	0.9600
C18A—H18A	0.9800	C23B—H23F	0.9600
C19A—C20A	1.519 (5)	C24B—H24D	0.9600
C19A—H19A	0.9700	C24B—H24E	0.9600
C19A—H19B	0.9700	C24B—H24F	0.9600
C20A—C21A	1.467 (5)	C21Y—C22Y	1.55 (4)
C20A—H20A	0.9700	C21Y—H21E	0.9700
C20A—H20B	0.9700	C21Y—H21F	0.9700
C21A—C22A	1.536 (5)	C22Y—C24Y	1.52 (4)
C21A—H21A	0.9700	C22Y—C23Y	1.62 (3)
C21A—H21B	0.9700	C22Y—H22C	0.9800
C22A—C24A	1.470 (6)	C23Y—H23G	0.9600
C22A—C23A	1.505 (6)	C23Y—H23H	0.9600
C22A—H22A	0.9800	C23Y—H23I	0.9600
C23A—H23A	0.9600	C24Y—H24G	0.9600
C23A—H23B	0.9600	C24Y—H24H	0.9600
C23A—H23C	0.9600	C24Y—H24I	0.9600
C24A—H24A	0.9600	C25B—H25D	0.9600
C24A—H24B	0.9600	C25B—H25E	0.9600
C24A—H24C	0.9600	C25B—H25F	0.9600
C25A—H25A	0.9600	C26B—H26D	0.9600
C25A—H25B	0.9600	C26B—H26E	0.9600
C25A—H25C	0.9600	C26B—H26F	0.9600
C26A—H26A	0.9600	C27B—H27D	0.9600
C26A—H26B	0.9600	C27B—H27E	0.9600
C26A—H26C	0.9600	C27B—H27F	0.9600
C27A—H27A	0.9600	C30B—C31B	1.543 (3)
C27A—H27B	0.9600	C30B—C46B	1.557 (3)
C27A—H27C	0.9600	C30B—H30C	0.9700
C30A—C31A	1.532 (3)	C30B—H30D	0.9700
C30A—C46A	1.557 (3)	C31B—C32B	1.515 (3)
C30A—H30A	0.9700	C31B—H31C	0.9700
C30A—H30B	0.9700	C31B—H31D	0.9700
C31A—C32A	1.525 (3)	C32B—C33B	1.523 (3)
C31A—H31A	0.9700	C32B—C45B	1.545 (3)
C31A—H31B	0.9700	C32B—H32B	0.9800
C32A—C33A	1.519 (3)	C33B—C42B	1.541 (3)
C32A—C45A	1.546 (3)	C33B—C34B	1.542 (3)
C32A—H32A	0.9800	C33B—H33B	0.9800
C33A—C42A	1.533 (3)	C34B—C35B	1.509 (3)
C33A—C34A	1.543 (3)	C34B—H34C	0.9700
C33A—H33A	0.9800	C34B—H34D	0.9700
C34A—C35A	1.501 (3)	C35B—C36B	1.505 (3)
C34A—H34A	0.9700	C36B—C37B	1.534 (3)
C34A—H34B	0.9700	C36B—C41B	1.561 (3)
C35A—C36A	1.516 (3)	C36B—H36B	0.9800
C36A—C37A	1.524 (3)	C37B—C38B	1.510 (3)
C36A—C41A	1.561 (3)	C37B—H37C	0.9700
C36A—H36A	0.9800	C37B—H37D	0.9700
C37A—C38A	1.523 (3)	C38B—C39B	1.511 (3)
C37A—H37A	0.9700	C38B—H38B	0.9800
C37A—H37B	0.9700	C39B—C40B	1.523 (3)
C38A—C39A	1.518 (4)	C39B—H39C	0.9700
C38A—H38A	0.9800	C39B—H39D	0.9700
C39A—C40A	1.532 (3)	C40B—C41B	1.540 (3)
C39A—H39A	0.9700	C40B—H40C	0.9700
C39A—H39B	0.9700	C40B—H40D	0.9700
C40A—C41A	1.542 (3)	C41B—C54B	1.536 (3)
C40A—H40A	0.9700	C41B—C42B	1.553 (3)
C40A—H40B	0.9700	C42B—C43B	1.538 (3)
C41A—C54A	1.534 (4)	C42B—H42B	0.9800
C41A—C42A	1.541 (3)	C43B—C44B	1.528 (3)
C42A—C43A	1.540 (3)	C43B—H43C	0.9700
C42A—H42A	0.9800	C43B—H43D	0.9700
C43A—C44A	1.539 (3)	C44B—C45B	1.536 (3)
C43A—H43A	0.9700	C44B—H44C	0.9700
C43A—H43B	0.9700	C44B—H44D	0.9700
C44A—C45A	1.530 (3)	C45B—C55B	1.541 (3)
C44A—H44A	0.9700	C45B—C46B	1.548 (3)
C44A—H44B	0.9700	C46B—C47B	1.542 (3)
C45A—C55A	1.535 (3)	C46B—H46B	0.9800
C45A—C46A	1.558 (3)	C47B—C56B	1.523 (4)
C46A—C47A	1.543 (3)	C47B—C48B	1.542 (3)
C46A—H46A	0.9800	C47B—H47B	0.9800
C47A—C56A	1.523 (4)	C48B—C49B	1.526 (4)
C47A—C48A	1.533 (3)	C48B—H48C	0.9700
C47A—H47A	0.9800	C48B—H48D	0.9700
C48A—C49A	1.512 (4)	C49B—C50B	1.512 (4)
C48A—H48A	0.9700	C49B—H49C	0.9700
C48A—H48B	0.9700	C49B—H49D	0.9700
C49A—C50A	1.524 (4)	C50B—C51B	1.534 (4)
C49A—H49A	0.9700	C50B—H50C	0.9700
C49A—H49B	0.9700	C50B—H50D	0.9700
C50A—C51A	1.528 (4)	C51B—C53B	1.524 (6)
C50A—H50A	0.9700	C51B—C52B	1.524 (5)
C50A—H50B	0.9700	C51B—H51B	0.9800
C51A—C52A	1.513 (4)	C52B—H52D	0.9600
C51A—C53A	1.514 (5)	C52B—H52E	0.9600
C51A—H51A	0.9800	C52B—H52F	0.9600
C52A—H52A	0.9600	C53B—H53D	0.9600
C52A—H52B	0.9600	C53B—H53E	0.9600
C52A—H52C	0.9600	C53B—H53F	0.9600
C53A—H53A	0.9600	C54B—H54D	0.9600
C53A—H53B	0.9600	C54B—H54E	0.9600
C53A—H53C	0.9600	C54B—H54F	0.9600
C54A—H54A	0.9600	C55B—H55D	0.9600
C54A—H54B	0.9600	C55B—H55E	0.9600
C54A—H54C	0.9600	C55B—H55F	0.9600
C55A—H55A	0.9600	C56B—H56D	0.9600
C55A—H55B	0.9600	C56B—H56E	0.9600
C55A—H55C	0.9600	C56B—H56F	0.9600
C56A—H56A	0.9600	C57B—C58B	1.519 (5)
C56A—H56B	0.9600	C58B—H58D	0.9600
C56A—H56C	0.9600	C58B—H58E	0.9600
C57A—C58A	1.498 (4)	C58B—H58F	0.9600
C58A—H58A	0.9600	O2A—C28A	1.189 (5)
C58A—H58B	0.9600	C28A—C29A	1.502 (5)
C58A—H58C	0.9600	C29A—H29A	0.9600
O1B—C28Y	1.23 (8)	C29A—H29B	0.9600
O1B—C28B	1.356 (5)	C29A—H29C	0.9600
O1B—C9B	1.446 (4)	O2B—C28B	1.205 (6)
O3B—C57B	1.347 (4)	C28B—C29B	1.503 (8)
O3B—C38B	1.455 (3)	C29B—H29D	0.9600
O4B—C57B	1.202 (5)	C29B—H29E	0.9600
N1B—C6B	1.279 (3)	C29B—H29F	0.9600
N1B—N2B	1.401 (3)	O2Y—C28Y	1.37 (10)
N2B—C35B	1.273 (3)	C28Y—C29Y	1.49 (12)
C1B—C2B	1.534 (4)	C29Y—H29G	0.9600
C1B—C17B	1.561 (4)	C29Y—H29H	0.9600
C1B—H1BA	0.9700	C29Y—H29I	0.9600
C1B—H1BB	0.9700	O2X—C28X	1.28 (9)
C2B—C3B	1.516 (4)	C28X—C29X	1.51 (14)
C2B—H2BA	0.9700	C29X—H29J	0.9600
C2B—H2BB	0.9700	C29X—H29K	0.9600
C3B—C4B	1.524 (3)	C29X—H29L	0.9600
C3B—C16B	1.543 (3)		
			
C28A—O1A—C9A	117.5 (2)	C3B—C4B—C5B	111.71 (19)
C28A—O1A—C28X	71 (2)	C3B—C4B—C13B	108.93 (18)
C9A—O1A—C28X	125 (3)	C5B—C4B—C13B	111.34 (19)
C57A—O3A—C38A	117.5 (2)	C3B—C4B—H4BA	108.3
C6A—N1A—N2A	116.92 (19)	C5B—C4B—H4BA	108.3
C35A—N2A—N1A	116.82 (19)	C13B—C4B—H4BA	108.3
C2A—C1A—C17A	107.1 (2)	C6B—C5B—C4B	109.80 (18)
C2A—C1A—H1AA	110.3	C6B—C5B—H5BA	109.7
C17A—C1A—H1AA	110.3	C4B—C5B—H5BA	109.7
C2A—C1A—H1AB	110.3	C6B—C5B—H5BB	109.7
C17A—C1A—H1AB	110.3	C4B—C5B—H5BB	109.7
H1AA—C1A—H1AB	108.5	H5BA—C5B—H5BB	108.2
C3A—C2A—C1A	104.2 (2)	N1B—C6B—C7B	119.5 (2)
C3A—C2A—H2AA	110.9	N1B—C6B—C5B	126.4 (2)
C1A—C2A—H2AA	110.9	C7B—C6B—C5B	113.95 (18)
C3A—C2A—H2AB	110.9	C6B—C7B—C8B	114.01 (19)
C1A—C2A—H2AB	110.9	C6B—C7B—C12B	110.35 (18)
H2AA—C2A—H2AB	108.9	C8B—C7B—C12B	113.2 (2)
C4A—C3A—C2A	118.3 (2)	C6B—C7B—H7BA	106.2
C4A—C3A—C16A	113.07 (19)	C8B—C7B—H7BA	106.2
C2A—C3A—C16A	104.57 (19)	C12B—C7B—H7BA	106.2
C4A—C3A—H3AA	106.8	C9B—C8B—C7B	109.8 (2)
C2A—C3A—H3AA	106.8	C9B—C8B—H8BA	109.7
C16A—C3A—H3AA	106.8	C7B—C8B—H8BA	109.7
C3A—C4A—C5A	112.24 (18)	C9B—C8B—H8BB	109.7
C3A—C4A—C13A	109.61 (18)	C7B—C8B—H8BB	109.7
C5A—C4A—C13A	110.62 (18)	H8BA—C8B—H8BB	108.2
C3A—C4A—H4AA	108.1	O1B—C9B—C10B	109.3 (2)
C5A—C4A—H4AA	108.1	O1B—C9B—C8B	107.0 (2)
C13A—C4A—H4AA	108.1	C10B—C9B—C8B	112.8 (2)
C6A—C5A—C4A	110.21 (18)	O1B—C9B—H9BA	109.2
C6A—C5A—H5AA	109.6	C10B—C9B—H9BA	109.2
C4A—C5A—H5AA	109.6	C8B—C9B—H9BA	109.2
C6A—C5A—H5AB	109.6	C9B—C10B—C11B	110.7 (2)
C4A—C5A—H5AB	109.6	C9B—C10B—H10C	109.5
H5AA—C5A—H5AB	108.1	C11B—C10B—H10C	109.5
N1A—C6A—C5A	126.8 (2)	C9B—C10B—H10D	109.5
N1A—C6A—C7A	119.6 (2)	C11B—C10B—H10D	109.5
C5A—C6A—C7A	113.4 (2)	H10C—C10B—H10D	108.1
C6A—C7A—C8A	113.8 (2)	C10B—C11B—C12B	113.3 (2)
C6A—C7A—C12A	110.31 (18)	C10B—C11B—H11C	108.9
C8A—C7A—C12A	113.3 (2)	C12B—C11B—H11C	108.9
C6A—C7A—H7AA	106.3	C10B—C11B—H11D	108.9
C8A—C7A—H7AA	106.3	C12B—C11B—H11D	108.9
C12A—C7A—H7AA	106.3	H11C—C11B—H11D	107.7
C9A—C8A—C7A	110.5 (2)	C25B—C12B—C11B	109.67 (19)
C9A—C8A—H8AA	109.5	C25B—C12B—C13B	110.78 (19)
C7A—C8A—H8AA	109.5	C11B—C12B—C13B	110.32 (18)
C9A—C8A—H8AB	109.5	C25B—C12B—C7B	110.93 (18)
C7A—C8A—H8AB	109.5	C11B—C12B—C7B	107.08 (18)
H8AA—C8A—H8AB	108.1	C13B—C12B—C7B	107.99 (18)
O1A—C9A—C10A	110.2 (2)	C14B—C13B—C4B	110.38 (19)
O1A—C9A—C8A	106.1 (2)	C14B—C13B—C12B	113.75 (19)
C10A—C9A—C8A	112.6 (2)	C4B—C13B—C12B	113.08 (17)
O1A—C9A—H9AA	109.3	C14B—C13B—H13B	106.3
C10A—C9A—H9AA	109.3	C4B—C13B—H13B	106.3
C8A—C9A—H9AA	109.3	C12B—C13B—H13B	106.3
C9A—C10A—C11A	110.7 (2)	C13B—C14B—C15B	113.7 (2)
C9A—C10A—H10A	109.5	C13B—C14B—H14C	108.8
C11A—C10A—H10A	109.5	C15B—C14B—H14C	108.8
C9A—C10A—H10B	109.5	C13B—C14B—H14D	108.8
C11A—C10A—H10B	109.5	C15B—C14B—H14D	108.8
H10A—C10A—H10B	108.1	H14C—C14B—H14D	107.7
C10A—C11A—C12A	113.5 (2)	C16B—C15B—C14B	111.5 (2)
C10A—C11A—H11A	108.9	C16B—C15B—H15C	109.3
C12A—C11A—H11A	108.9	C14B—C15B—H15C	109.3
C10A—C11A—H11B	108.9	C16B—C15B—H15D	109.3
C12A—C11A—H11B	108.9	C14B—C15B—H15D	109.3
H11A—C11A—H11B	107.7	H15C—C15B—H15D	108.0
C25A—C12A—C11A	109.43 (19)	C15B—C16B—C3B	107.10 (19)
C25A—C12A—C13A	111.16 (19)	C15B—C16B—C26B	110.8 (2)
C11A—C12A—C13A	110.32 (19)	C3B—C16B—C26B	111.9 (2)
C25A—C12A—C7A	110.5 (2)	C15B—C16B—C17B	116.53 (19)
C11A—C12A—C7A	107.4 (2)	C3B—C16B—C17B	99.69 (19)
C13A—C12A—C7A	107.95 (18)	C26B—C16B—C17B	110.4 (2)
C14A—C13A—C4A	112.44 (19)	C18B—C17B—C1B	111.2 (2)
C14A—C13A—C12A	113.76 (19)	C18B—C17B—C16B	119.9 (2)
C4A—C13A—C12A	111.64 (18)	C1B—C17B—C16B	103.62 (19)
C14A—C13A—H13A	106.1	C18B—C17B—H17B	107.2
C4A—C13A—H13A	106.1	C1B—C17B—H17B	107.2
C12A—C13A—H13A	106.1	C16B—C17B—H17B	107.2
C13A—C14A—C15A	113.9 (2)	C27B—C18B—C17B	113.5 (3)
C13A—C14A—H14A	108.8	C27B—C18B—C19B	110.1 (3)
C15A—C14A—H14A	108.8	C17B—C18B—C19B	110.1 (3)
C13A—C14A—H14B	108.8	C27B—C18B—H18B	107.6
C15A—C14A—H14B	108.8	C17B—C18B—H18B	107.6
H14A—C14A—H14B	107.7	C19B—C18B—H18B	107.6
C16A—C15A—C14A	111.8 (2)	C20B—C19B—C18B	116.9 (3)
C16A—C15A—H15A	109.3	C20B—C19B—H19C	108.1
C14A—C15A—H15A	109.3	C18B—C19B—H19C	108.1
C16A—C15A—H15B	109.3	C20B—C19B—H19D	108.1
C14A—C15A—H15B	109.3	C18B—C19B—H19D	108.1
H15A—C15A—H15B	107.9	H19C—C19B—H19D	107.3
C3A—C16A—C15A	107.36 (19)	C21Y—C20B—C19B	131.8 (11)
C3A—C16A—C26A	111.84 (19)	C19B—C20B—C21B	112.0 (4)
C15A—C16A—C26A	109.2 (2)	C21Y—C20B—H20C	113.0
C3A—C16A—C17A	100.78 (18)	C19B—C20B—H20C	109.2
C15A—C16A—C17A	116.82 (19)	C21B—C20B—H20C	109.2
C26A—C16A—C17A	110.6 (2)	C21Y—C20B—H20D	78.8
C16A—C17A—C18A	119.0 (2)	C19B—C20B—H20D	109.2
C16A—C17A—C1A	101.6 (2)	C21B—C20B—H20D	109.2
C18A—C17A—C1A	115.0 (2)	H20C—C20B—H20D	107.9
C16A—C17A—H17A	106.9	C21Y—C20B—H20E	104.2
C18A—C17A—H17A	106.9	C19B—C20B—H20E	105.4
C1A—C17A—H17A	106.9	C21B—C20B—H20E	90.5
C19A—C18A—C27A	109.8 (2)	H20D—C20B—H20E	129.0
C19A—C18A—C17A	115.3 (2)	C21Y—C20B—H20F	103.3
C27A—C18A—C17A	109.2 (2)	C19B—C20B—H20F	104.2
C19A—C18A—H18A	107.4	C21B—C20B—H20F	134.6
C27A—C18A—H18A	107.4	H20C—C20B—H20F	82.4
C17A—C18A—H18A	107.4	H20E—C20B—H20F	105.6
C18A—C19A—C20A	116.2 (3)	C22B—C21B—C20B	114.3 (4)
C18A—C19A—H19A	108.2	C22B—C21B—H21C	108.7
C20A—C19A—H19A	108.2	C20B—C21B—H21C	108.7
C18A—C19A—H19B	108.2	C22B—C21B—H21D	108.7
C20A—C19A—H19B	108.2	C20B—C21B—H21D	108.7
H19A—C19A—H19B	107.4	H21C—C21B—H21D	107.6
C21A—C20A—C19A	115.2 (3)	C24B—C22B—C21B	112.8 (5)
C21A—C20A—H20A	108.5	C24B—C22B—C23B	111.0 (6)
C19A—C20A—H20A	108.5	C21B—C22B—C23B	110.5 (5)
C21A—C20A—H20B	108.5	C24B—C22B—H22B	107.5
C19A—C20A—H20B	108.5	C21B—C22B—H22B	107.5
H20A—C20A—H20B	107.5	C23B—C22B—H22B	107.5
C20A—C21A—C22A	117.1 (3)	C20B—C21Y—C22Y	99.4 (17)
C20A—C21A—H21A	108.0	C20B—C21Y—H21E	111.9
C22A—C21A—H21A	108.0	C22Y—C21Y—H21E	111.9
C20A—C21A—H21B	108.0	C20B—C21Y—H21F	111.9
C22A—C21A—H21B	108.0	C22Y—C21Y—H21F	111.9
H21A—C21A—H21B	107.3	H21E—C21Y—H21F	109.6
C24A—C22A—C23A	112.4 (3)	C24Y—C22Y—C21Y	118 (2)
C24A—C22A—C21A	113.7 (3)	C24Y—C22Y—C23Y	105 (2)
C23A—C22A—C21A	111.3 (4)	C21Y—C22Y—C23Y	107 (2)
C24A—C22A—H22A	106.3	C24Y—C22Y—H22C	109.0
C23A—C22A—H22A	106.3	C21Y—C22Y—H22C	109.0
C21A—C22A—H22A	106.3	C23Y—C22Y—H22C	109.0
C22A—C23A—H23A	109.5	C22Y—C23Y—H23G	109.5
C22A—C23A—H23B	109.5	C22Y—C23Y—H23H	109.5
H23A—C23A—H23B	109.5	H23G—C23Y—H23H	109.5
C22A—C23A—H23C	109.5	C22Y—C23Y—H23I	109.5
H23A—C23A—H23C	109.5	H23G—C23Y—H23I	109.5
H23B—C23A—H23C	109.5	H23H—C23Y—H23I	109.5
C22A—C24A—H24A	109.5	C22Y—C24Y—H24G	109.5
C22A—C24A—H24B	109.5	C22Y—C24Y—H24H	109.5
H24A—C24A—H24B	109.5	H24G—C24Y—H24H	109.5
C22A—C24A—H24C	109.5	C22Y—C24Y—H24I	109.5
H24A—C24A—H24C	109.5	H24G—C24Y—H24I	109.5
H24B—C24A—H24C	109.5	H24H—C24Y—H24I	109.5
C12A—C25A—H25A	109.5	C12B—C25B—H25D	109.5
C12A—C25A—H25B	109.5	C12B—C25B—H25E	109.5
H25A—C25A—H25B	109.5	H25D—C25B—H25E	109.5
C12A—C25A—H25C	109.5	C12B—C25B—H25F	109.5
H25A—C25A—H25C	109.5	H25D—C25B—H25F	109.5
H25B—C25A—H25C	109.5	H25E—C25B—H25F	109.5
C16A—C26A—H26A	109.5	C16B—C26B—H26D	109.5
C16A—C26A—H26B	109.5	C16B—C26B—H26E	109.5
H26A—C26A—H26B	109.5	H26D—C26B—H26E	109.5
C16A—C26A—H26C	109.5	C16B—C26B—H26F	109.5
H26A—C26A—H26C	109.5	H26D—C26B—H26F	109.5
H26B—C26A—H26C	109.5	H26E—C26B—H26F	109.5
C18A—C27A—H27A	109.5	C18B—C27B—H27D	109.5
C18A—C27A—H27B	109.5	C18B—C27B—H27E	109.5
H27A—C27A—H27B	109.5	H27D—C27B—H27E	109.5
C18A—C27A—H27C	109.5	C18B—C27B—H27F	109.5
H27A—C27A—H27C	109.5	H27D—C27B—H27F	109.5
H27B—C27A—H27C	109.5	H27E—C27B—H27F	109.5
C31A—C30A—C46A	107.37 (18)	C31B—C30B—C46B	106.63 (19)
C31A—C30A—H30A	110.2	C31B—C30B—H30C	110.4
C46A—C30A—H30A	110.2	C46B—C30B—H30C	110.4
C31A—C30A—H30B	110.2	C31B—C30B—H30D	110.4
C46A—C30A—H30B	110.2	C46B—C30B—H30D	110.4
H30A—C30A—H30B	108.5	H30C—C30B—H30D	108.6
C32A—C31A—C30A	104.55 (18)	C32B—C31B—C30B	103.47 (18)
C32A—C31A—H31A	110.8	C32B—C31B—H31C	111.1
C30A—C31A—H31A	110.8	C30B—C31B—H31C	111.1
C32A—C31A—H31B	110.8	C32B—C31B—H31D	111.1
C30A—C31A—H31B	110.8	C30B—C31B—H31D	111.1
H31A—C31A—H31B	108.9	H31C—C31B—H31D	109.0
C33A—C32A—C31A	119.19 (18)	C31B—C32B—C33B	119.27 (18)
C33A—C32A—C45A	114.56 (18)	C31B—C32B—C45B	103.55 (17)
C31A—C32A—C45A	103.86 (19)	C33B—C32B—C45B	115.26 (18)
C33A—C32A—H32A	106.1	C31B—C32B—H32B	105.9
C31A—C32A—H32A	106.1	C33B—C32B—H32B	105.9
C45A—C32A—H32A	106.1	C45B—C32B—H32B	105.9
C32A—C33A—C42A	109.08 (17)	C32B—C33B—C42B	109.26 (16)
C32A—C33A—C34A	111.59 (18)	C32B—C33B—C34B	110.31 (18)
C42A—C33A—C34A	110.38 (18)	C42B—C33B—C34B	111.13 (16)
C32A—C33A—H33A	108.6	C32B—C33B—H33B	108.7
C42A—C33A—H33A	108.6	C42B—C33B—H33B	108.7
C34A—C33A—H33A	108.6	C34B—C33B—H33B	108.7
C35A—C34A—C33A	110.90 (18)	C35B—C34B—C33B	111.63 (17)
C35A—C34A—H34A	109.5	C35B—C34B—H34C	109.3
C33A—C34A—H34A	109.5	C33B—C34B—H34C	109.3
C35A—C34A—H34B	109.5	C35B—C34B—H34D	109.3
C33A—C34A—H34B	109.5	C33B—C34B—H34D	109.3
H34A—C34A—H34B	108.0	H34C—C34B—H34D	108.0
N2A—C35A—C34A	126.8 (2)	N2B—C35B—C36B	119.12 (19)
N2A—C35A—C36A	119.7 (2)	N2B—C35B—C34B	126.4 (2)
C34A—C35A—C36A	113.46 (18)	C36B—C35B—C34B	114.45 (17)
C35A—C36A—C37A	114.68 (18)	C35B—C36B—C37B	113.89 (17)
C35A—C36A—C41A	109.19 (18)	C35B—C36B—C41B	110.79 (17)
C37A—C36A—C41A	113.06 (19)	C37B—C36B—C41B	113.40 (18)
C35A—C36A—H36A	106.4	C35B—C36B—H36B	106.0
C37A—C36A—H36A	106.4	C37B—C36B—H36B	106.0
C41A—C36A—H36A	106.4	C41B—C36B—H36B	106.0
C38A—C37A—C36A	110.35 (18)	C38B—C37B—C36B	109.11 (18)
C38A—C37A—H37A	109.6	C38B—C37B—H37C	109.9
C36A—C37A—H37A	109.6	C36B—C37B—H37C	109.9
C38A—C37A—H37B	109.6	C38B—C37B—H37D	109.9
C36A—C37A—H37B	109.6	C36B—C37B—H37D	109.9
H37A—C37A—H37B	108.1	H37C—C37B—H37D	108.3
O3A—C38A—C39A	106.15 (19)	O3B—C38B—C37B	107.27 (18)
O3A—C38A—C37A	111.14 (18)	O3B—C38B—C39B	109.2 (2)
C39A—C38A—C37A	110.9 (2)	C37B—C38B—C39B	111.78 (19)
O3A—C38A—H38A	109.5	O3B—C38B—H38B	109.5
C39A—C38A—H38A	109.5	C37B—C38B—H38B	109.5
C37A—C38A—H38A	109.5	C39B—C38B—H38B	109.5
C38A—C39A—C40A	110.9 (2)	C38B—C39B—C40B	110.9 (2)
C38A—C39A—H39A	109.5	C38B—C39B—H39C	109.5
C40A—C39A—H39A	109.5	C40B—C39B—H39C	109.5
C38A—C39A—H39B	109.5	C38B—C39B—H39D	109.5
C40A—C39A—H39B	109.5	C40B—C39B—H39D	109.5
H39A—C39A—H39B	108.1	H39C—C39B—H39D	108.0
C39A—C40A—C41A	113.91 (19)	C39B—C40B—C41B	113.63 (18)
C39A—C40A—H40A	108.8	C39B—C40B—H40C	108.8
C41A—C40A—H40A	108.8	C41B—C40B—H40C	108.8
C39A—C40A—H40B	108.8	C39B—C40B—H40D	108.8
C41A—C40A—H40B	108.8	C41B—C40B—H40D	108.8
H40A—C40A—H40B	107.7	H40C—C40B—H40D	107.7
C54A—C41A—C42A	111.33 (19)	C54B—C41B—C40B	110.33 (18)
C54A—C41A—C40A	110.47 (19)	C54B—C41B—C42B	111.10 (17)
C42A—C41A—C40A	109.55 (18)	C40B—C41B—C42B	109.93 (17)
C54A—C41A—C36A	110.86 (18)	C54B—C41B—C36B	110.69 (17)
C42A—C41A—C36A	106.64 (18)	C40B—C41B—C36B	107.33 (17)
C40A—C41A—C36A	107.87 (19)	C42B—C41B—C36B	107.35 (17)
C33A—C42A—C43A	111.88 (19)	C43B—C42B—C33B	110.75 (17)
C33A—C42A—C41A	112.73 (17)	C43B—C42B—C41B	114.82 (18)
C43A—C42A—C41A	113.24 (18)	C33B—C42B—C41B	112.32 (16)
C33A—C42A—H42A	106.1	C43B—C42B—H42B	106.1
C43A—C42A—H42A	106.1	C33B—C42B—H42B	106.1
C41A—C42A—H42A	106.1	C41B—C42B—H42B	106.1
C44A—C43A—C42A	112.90 (19)	C44B—C43B—C42B	113.32 (19)
C44A—C43A—H43A	109.0	C44B—C43B—H43C	108.9
C42A—C43A—H43A	109.0	C42B—C43B—H43C	108.9
C44A—C43A—H43B	109.0	C44B—C43B—H43D	108.9
C42A—C43A—H43B	109.0	C42B—C43B—H43D	108.9
H43A—C43A—H43B	107.8	H43C—C43B—H43D	107.7
C45A—C44A—C43A	111.37 (19)	C43B—C44B—C45B	112.10 (18)
C45A—C44A—H44A	109.4	C43B—C44B—H44C	109.2
C43A—C44A—H44A	109.4	C45B—C44B—H44C	109.2
C45A—C44A—H44B	109.4	C43B—C44B—H44D	109.2
C43A—C44A—H44B	109.4	C45B—C44B—H44D	109.2
H44A—C44A—H44B	108.0	H44C—C44B—H44D	107.9
C44A—C45A—C55A	110.2 (2)	C44B—C45B—C55B	110.22 (19)
C44A—C45A—C32A	106.81 (18)	C44B—C45B—C32B	106.96 (17)
C55A—C45A—C32A	112.24 (19)	C55B—C45B—C32B	112.07 (17)
C44A—C45A—C46A	116.42 (19)	C44B—C45B—C46B	116.76 (17)
C55A—C45A—C46A	110.54 (19)	C55B—C45B—C46B	110.63 (18)
C32A—C45A—C46A	100.26 (17)	C32B—C45B—C46B	99.75 (17)
C47A—C46A—C30A	115.31 (19)	C47B—C46B—C45B	120.42 (19)
C47A—C46A—C45A	118.04 (19)	C47B—C46B—C30B	110.61 (19)
C30A—C46A—C45A	101.88 (17)	C45B—C46B—C30B	103.67 (17)
C47A—C46A—H46A	107.0	C47B—C46B—H46B	107.1
C30A—C46A—H46A	107.0	C45B—C46B—H46B	107.1
C45A—C46A—H46A	107.0	C30B—C46B—H46B	107.1
C56A—C47A—C48A	109.9 (2)	C56B—C47B—C46B	113.6 (2)
C56A—C47A—C46A	110.9 (2)	C56B—C47B—C48B	110.47 (19)
C48A—C47A—C46A	113.0 (2)	C46B—C47B—C48B	110.15 (19)
C56A—C47A—H47A	107.6	C56B—C47B—H47B	107.5
C48A—C47A—H47A	107.6	C46B—C47B—H47B	107.5
C46A—C47A—H47A	107.6	C48B—C47B—H47B	107.5
C49A—C48A—C47A	114.0 (2)	C49B—C48B—C47B	114.1 (2)
C49A—C48A—H48A	108.8	C49B—C48B—H48C	108.7
C47A—C48A—H48A	108.8	C47B—C48B—H48C	108.7
C49A—C48A—H48B	108.8	C49B—C48B—H48D	108.7
C47A—C48A—H48B	108.8	C47B—C48B—H48D	108.7
H48A—C48A—H48B	107.6	H48C—C48B—H48D	107.6
C48A—C49A—C50A	113.6 (2)	C50B—C49B—C48B	113.9 (2)
C48A—C49A—H49A	108.8	C50B—C49B—H49C	108.8
C50A—C49A—H49A	108.8	C48B—C49B—H49C	108.8
C48A—C49A—H49B	108.8	C50B—C49B—H49D	108.8
C50A—C49A—H49B	108.8	C48B—C49B—H49D	108.8
H49A—C49A—H49B	107.7	H49C—C49B—H49D	107.7
C49A—C50A—C51A	115.4 (2)	C49B—C50B—C51B	113.2 (3)
C49A—C50A—H50A	108.4	C49B—C50B—H50C	108.9
C51A—C50A—H50A	108.4	C51B—C50B—H50C	108.9
C49A—C50A—H50B	108.4	C49B—C50B—H50D	108.9
C51A—C50A—H50B	108.4	C51B—C50B—H50D	108.9
H50A—C50A—H50B	107.5	H50C—C50B—H50D	107.7
C52A—C51A—C53A	110.2 (3)	C53B—C51B—C52B	111.1 (3)
C52A—C51A—C50A	110.5 (3)	C53B—C51B—C50B	111.8 (3)
C53A—C51A—C50A	112.3 (2)	C52B—C51B—C50B	110.7 (3)
C52A—C51A—H51A	107.9	C53B—C51B—H51B	107.7
C53A—C51A—H51A	107.9	C52B—C51B—H51B	107.7
C50A—C51A—H51A	107.9	C50B—C51B—H51B	107.7
C51A—C52A—H52A	109.5	C51B—C52B—H52D	109.5
C51A—C52A—H52B	109.5	C51B—C52B—H52E	109.5
H52A—C52A—H52B	109.5	H52D—C52B—H52E	109.5
C51A—C52A—H52C	109.5	C51B—C52B—H52F	109.5
H52A—C52A—H52C	109.5	H52D—C52B—H52F	109.5
H52B—C52A—H52C	109.5	H52E—C52B—H52F	109.5
C51A—C53A—H53A	109.5	C51B—C53B—H53D	109.5
C51A—C53A—H53B	109.5	C51B—C53B—H53E	109.5
H53A—C53A—H53B	109.5	H53D—C53B—H53E	109.5
C51A—C53A—H53C	109.5	C51B—C53B—H53F	109.5
H53A—C53A—H53C	109.5	H53D—C53B—H53F	109.5
H53B—C53A—H53C	109.5	H53E—C53B—H53F	109.5
C41A—C54A—H54A	109.5	C41B—C54B—H54D	109.5
C41A—C54A—H54B	109.5	C41B—C54B—H54E	109.5
H54A—C54A—H54B	109.5	H54D—C54B—H54E	109.5
C41A—C54A—H54C	109.5	C41B—C54B—H54F	109.5
H54A—C54A—H54C	109.5	H54D—C54B—H54F	109.5
H54B—C54A—H54C	109.5	H54E—C54B—H54F	109.5
C45A—C55A—H55A	109.5	C45B—C55B—H55D	109.5
C45A—C55A—H55B	109.5	C45B—C55B—H55E	109.5
H55A—C55A—H55B	109.5	H55D—C55B—H55E	109.5
C45A—C55A—H55C	109.5	C45B—C55B—H55F	109.5
H55A—C55A—H55C	109.5	H55D—C55B—H55F	109.5
H55B—C55A—H55C	109.5	H55E—C55B—H55F	109.5
C47A—C56A—H56A	109.5	C47B—C56B—H56D	109.5
C47A—C56A—H56B	109.5	C47B—C56B—H56E	109.5
H56A—C56A—H56B	109.5	H56D—C56B—H56E	109.5
C47A—C56A—H56C	109.5	C47B—C56B—H56F	109.5
H56A—C56A—H56C	109.5	H56D—C56B—H56F	109.5
H56B—C56A—H56C	109.5	H56E—C56B—H56F	109.5
O4A—C57A—O3A	123.8 (3)	O4B—C57B—O3B	123.7 (3)
O4A—C57A—C58A	124.7 (3)	O4B—C57B—C58B	127.0 (3)
O3A—C57A—C58A	111.4 (3)	O3B—C57B—C58B	109.2 (3)
C57A—C58A—H58A	109.5	C57B—C58B—H58D	109.5
C57A—C58A—H58B	109.5	C57B—C58B—H58E	109.5
H58A—C58A—H58B	109.5	H58D—C58B—H58E	109.5
C57A—C58A—H58C	109.5	C57B—C58B—H58F	109.5
H58A—C58A—H58C	109.5	H58D—C58B—H58F	109.5
H58B—C58A—H58C	109.5	H58E—C58B—H58F	109.5
C28Y—O1B—C28B	73 (4)	O2A—C28A—O1A	124.6 (3)
C28Y—O1B—C9B	146 (4)	O2A—C28A—C29A	125.1 (3)
C28B—O1B—C9B	115.5 (3)	O1A—C28A—C29A	110.3 (3)
C57B—O3B—C38B	116.3 (2)	O2B—C28B—O1B	125.1 (4)
C6B—N1B—N2B	116.87 (19)	O2B—C28B—C29B	126.8 (5)
C35B—N2B—N1B	116.93 (18)	O1B—C28B—C29B	108.0 (5)
C2B—C1B—C17B	107.2 (2)	O1B—C28Y—O2Y	102 (7)
C2B—C1B—H1BA	110.3	O1B—C28Y—C29Y	129 (8)
C17B—C1B—H1BA	110.3	O2Y—C28Y—C29Y	121 (7)
C2B—C1B—H1BB	110.3	C28Y—C29Y—H29G	109.5
C17B—C1B—H1BB	110.3	C28Y—C29Y—H29H	109.5
H1BA—C1B—H1BB	108.5	H29G—C29Y—H29H	109.5
C3B—C2B—C1B	103.6 (2)	C28Y—C29Y—H29I	109.5
C3B—C2B—H2BA	111.0	H29G—C29Y—H29I	109.5
C1B—C2B—H2BA	111.0	H29H—C29Y—H29I	109.5
C3B—C2B—H2BB	111.0	O2X—C28X—C29X	149 (7)
C1B—C2B—H2BB	111.0	O2X—C28X—O1A	110 (6)
H2BA—C2B—H2BB	109.0	C29X—C28X—O1A	96 (6)
C2B—C3B—C4B	119.19 (19)	C28X—C29X—H29J	109.5
C2B—C3B—C16B	104.5 (2)	C28X—C29X—H29K	109.5
C4B—C3B—C16B	114.08 (19)	H29J—C29X—H29K	109.5
C2B—C3B—H3BA	106.0	C28X—C29X—H29L	109.5
C4B—C3B—H3BA	106.0	H29J—C29X—H29L	109.5
C16B—C3B—H3BA	106.0	H29K—C29X—H29L	109.5
			
C6A—N1A—N2A—C35A	120.8 (2)	N2B—N1B—C6B—C5B	−6.4 (3)
C17A—C1A—C2A—C3A	−0.5 (3)	C4B—C5B—C6B—N1B	−118.2 (2)
C1A—C2A—C3A—C4A	−154.3 (2)	C4B—C5B—C6B—C7B	57.3 (2)
C1A—C2A—C3A—C16A	−27.5 (3)	N1B—C6B—C7B—C8B	−13.0 (3)
C2A—C3A—C4A—C5A	−54.8 (3)	C5B—C6B—C7B—C8B	171.27 (19)
C16A—C3A—C4A—C5A	−177.44 (17)	N1B—C6B—C7B—C12B	115.7 (2)
C2A—C3A—C4A—C13A	−178.1 (2)	C5B—C6B—C7B—C12B	−60.1 (2)
C16A—C3A—C4A—C13A	59.2 (2)	C6B—C7B—C8B—C9B	−176.3 (2)
C3A—C4A—C5A—C6A	−176.92 (17)	C12B—C7B—C8B—C9B	56.5 (3)
C13A—C4A—C5A—C6A	−54.2 (2)	C28Y—O1B—C9B—C10B	−171 (7)
N2A—N1A—C6A—C5A	−7.9 (3)	C28B—O1B—C9B—C10B	91.7 (4)
N2A—N1A—C6A—C7A	178.26 (19)	C28Y—O1B—C9B—C8B	−49 (7)
C4A—C5A—C6A—N1A	−117.2 (2)	C28B—O1B—C9B—C8B	−146.0 (3)
C4A—C5A—C6A—C7A	57.0 (2)	C7B—C8B—C9B—O1B	−175.4 (2)
N1A—C6A—C7A—C8A	−13.5 (3)	C7B—C8B—C9B—C10B	−55.2 (3)
C5A—C6A—C7A—C8A	171.93 (19)	O1B—C9B—C10B—C11B	173.8 (2)
N1A—C6A—C7A—C12A	115.2 (2)	C8B—C9B—C10B—C11B	54.9 (3)
C5A—C6A—C7A—C12A	−59.4 (2)	C9B—C10B—C11B—C12B	−56.3 (3)
C6A—C7A—C8A—C9A	−177.0 (2)	C10B—C11B—C12B—C25B	−65.1 (3)
C12A—C7A—C8A—C9A	55.8 (3)	C10B—C11B—C12B—C13B	172.6 (2)
C28A—O1A—C9A—C10A	92.2 (3)	C10B—C11B—C12B—C7B	55.3 (3)
C28X—O1A—C9A—C10A	178 (2)	C6B—C7B—C12B—C25B	−65.5 (2)
C28A—O1A—C9A—C8A	−145.6 (3)	C8B—C7B—C12B—C25B	63.6 (3)
C28X—O1A—C9A—C8A	−60 (2)	C6B—C7B—C12B—C11B	174.85 (18)
C7A—C8A—C9A—O1A	−175.9 (2)	C8B—C7B—C12B—C11B	−56.0 (2)
C7A—C8A—C9A—C10A	−55.2 (3)	C6B—C7B—C12B—C13B	56.1 (2)
O1A—C9A—C10A—C11A	173.1 (2)	C8B—C7B—C12B—C13B	−174.82 (17)
C8A—C9A—C10A—C11A	54.9 (3)	C3B—C4B—C13B—C14B	−53.6 (2)
C9A—C10A—C11A—C12A	−55.9 (3)	C5B—C4B—C13B—C14B	−177.26 (18)
C10A—C11A—C12A—C25A	−65.6 (3)	C3B—C4B—C13B—C12B	177.66 (17)
C10A—C11A—C12A—C13A	171.8 (2)	C5B—C4B—C13B—C12B	54.0 (2)
C10A—C11A—C12A—C7A	54.4 (3)	C25B—C12B—C13B—C14B	−59.9 (2)
C6A—C7A—C12A—C25A	−64.3 (2)	C11B—C12B—C13B—C14B	61.7 (2)
C8A—C7A—C12A—C25A	64.6 (2)	C7B—C12B—C13B—C14B	178.40 (18)
C6A—C7A—C12A—C11A	176.39 (17)	C25B—C12B—C13B—C4B	67.0 (2)
C8A—C7A—C12A—C11A	−54.7 (2)	C11B—C12B—C13B—C4B	−171.36 (18)
C6A—C7A—C12A—C13A	57.5 (2)	C7B—C12B—C13B—C4B	−54.7 (2)
C8A—C7A—C12A—C13A	−173.61 (17)	C4B—C13B—C14B—C15B	53.6 (3)
C3A—C4A—C13A—C14A	−50.2 (2)	C12B—C13B—C14B—C15B	−178.11 (18)
C5A—C4A—C13A—C14A	−174.51 (19)	C13B—C14B—C15B—C16B	−55.4 (3)
C3A—C4A—C13A—C12A	−179.48 (17)	C14B—C15B—C16B—C3B	55.2 (2)
C5A—C4A—C13A—C12A	56.2 (2)	C14B—C15B—C16B—C26B	−67.0 (3)
C25A—C12A—C13A—C14A	−64.2 (3)	C14B—C15B—C16B—C17B	165.7 (2)
C11A—C12A—C13A—C14A	57.4 (3)	C2B—C3B—C16B—C15B	168.43 (18)
C7A—C12A—C13A—C14A	174.39 (18)	C4B—C3B—C16B—C15B	−59.7 (2)
C25A—C12A—C13A—C4A	64.3 (3)	C2B—C3B—C16B—C26B	−70.0 (2)
C11A—C12A—C13A—C4A	−174.07 (19)	C4B—C3B—C16B—C26B	61.9 (3)
C7A—C12A—C13A—C4A	−57.0 (2)	C2B—C3B—C16B—C17B	46.7 (2)
C4A—C13A—C14A—C15A	47.9 (3)	C4B—C3B—C16B—C17B	178.55 (18)
C12A—C13A—C14A—C15A	176.03 (18)	C2B—C1B—C17B—C18B	146.6 (2)
C13A—C14A—C15A—C16A	−51.8 (3)	C2B—C1B—C17B—C16B	16.5 (3)
C4A—C3A—C16A—C15A	−62.3 (2)	C15B—C16B—C17B—C18B	83.0 (3)
C2A—C3A—C16A—C15A	167.76 (19)	C3B—C16B—C17B—C18B	−162.2 (2)
C4A—C3A—C16A—C26A	57.5 (3)	C26B—C16B—C17B—C18B	−44.4 (3)
C2A—C3A—C16A—C26A	−72.5 (3)	C15B—C16B—C17B—C1B	−152.4 (2)
C4A—C3A—C16A—C17A	175.00 (17)	C3B—C16B—C17B—C1B	−37.7 (2)
C2A—C3A—C16A—C17A	45.0 (2)	C26B—C16B—C17B—C1B	80.2 (3)
C14A—C15A—C16A—C3A	56.5 (2)	C1B—C17B—C18B—C27B	−175.7 (3)
C14A—C15A—C16A—C26A	−64.9 (3)	C16B—C17B—C18B—C27B	−54.8 (3)
C14A—C15A—C16A—C17A	168.7 (2)	C1B—C17B—C18B—C19B	60.3 (3)
C3A—C16A—C17A—C18A	−171.1 (2)	C16B—C17B—C18B—C19B	−178.8 (2)
C15A—C16A—C17A—C18A	73.0 (3)	C27B—C18B—C19B—C20B	78.5 (4)
C26A—C16A—C17A—C18A	−52.7 (3)	C17B—C18B—C19B—C20B	−155.6 (3)
C3A—C16A—C17A—C1A	−43.8 (2)	C18B—C19B—C20B—C21Y	−157.4 (15)
C15A—C16A—C17A—C1A	−159.7 (2)	C18B—C19B—C20B—C21B	173.6 (4)
C26A—C16A—C17A—C1A	74.6 (2)	C21Y—C20B—C21B—C22B	54 (2)
C2A—C1A—C17A—C16A	27.7 (3)	C19B—C20B—C21B—C22B	−169.8 (4)
C2A—C1A—C17A—C18A	157.6 (2)	C20B—C21B—C22B—C24B	68.4 (7)
C16A—C17A—C18A—C19A	152.6 (2)	C20B—C21B—C22B—C23B	−166.8 (6)
C1A—C17A—C18A—C19A	31.9 (3)	C19B—C20B—C21Y—C22Y	−87.5 (17)
C16A—C17A—C18A—C27A	−83.4 (3)	C21B—C20B—C21Y—C22Y	−27.6 (13)
C1A—C17A—C18A—C27A	155.9 (3)	C20B—C21Y—C22Y—C24Y	−76 (2)
C27A—C18A—C19A—C20A	56.7 (4)	C20B—C21Y—C22Y—C23Y	166.1 (18)
C17A—C18A—C19A—C20A	−179.6 (2)	C46B—C30B—C31B—C32B	12.9 (2)
C18A—C19A—C20A—C21A	160.4 (3)	C30B—C31B—C32B—C33B	−167.72 (18)
C19A—C20A—C21A—C22A	−177.9 (3)	C30B—C31B—C32B—C45B	−38.1 (2)
C20A—C21A—C22A—C24A	−65.4 (5)	C31B—C32B—C33B—C42B	−178.26 (17)
C20A—C21A—C22A—C23A	166.4 (4)	C45B—C32B—C33B—C42B	57.6 (2)
C46A—C30A—C31A—C32A	4.2 (3)	C31B—C32B—C33B—C34B	−55.8 (2)
C30A—C31A—C32A—C33A	−160.3 (2)	C45B—C32B—C33B—C34B	−179.98 (16)
C30A—C31A—C32A—C45A	−31.4 (2)	C32B—C33B—C34B—C35B	−171.46 (16)
C31A—C32A—C33A—C42A	−178.1 (2)	C42B—C33B—C34B—C35B	−50.1 (2)
C45A—C32A—C33A—C42A	58.1 (2)	N1B—N2B—C35B—C36B	174.71 (17)
C31A—C32A—C33A—C34A	−55.9 (3)	N1B—N2B—C35B—C34B	−4.5 (3)
C45A—C32A—C33A—C34A	−179.72 (18)	C33B—C34B—C35B—N2B	−128.7 (2)
C32A—C33A—C34A—C35A	−172.98 (19)	C33B—C34B—C35B—C36B	52.1 (2)
C42A—C33A—C34A—C35A	−51.5 (2)	N2B—C35B—C36B—C37B	−5.2 (3)
N1A—N2A—C35A—C34A	−6.1 (3)	C34B—C35B—C36B—C37B	174.06 (17)
N1A—N2A—C35A—C36A	176.93 (19)	N2B—C35B—C36B—C41B	124.0 (2)
C33A—C34A—C35A—N2A	−121.9 (2)	C34B—C35B—C36B—C41B	−56.7 (2)
C33A—C34A—C35A—C36A	55.2 (3)	C35B—C36B—C37B—C38B	−174.32 (18)
N2A—C35A—C36A—C37A	−10.7 (3)	C41B—C36B—C37B—C38B	57.8 (2)
C34A—C35A—C36A—C37A	171.9 (2)	C57B—O3B—C38B—C37B	−155.4 (2)
N2A—C35A—C36A—C41A	117.3 (2)	C57B—O3B—C38B—C39B	83.3 (3)
C34A—C35A—C36A—C41A	−60.0 (3)	C36B—C37B—C38B—O3B	−177.22 (18)
C35A—C36A—C37A—C38A	−176.3 (2)	C36B—C37B—C38B—C39B	−57.6 (2)
C41A—C36A—C37A—C38A	57.6 (3)	O3B—C38B—C39B—C40B	175.52 (18)
C57A—O3A—C38A—C39A	146.1 (2)	C37B—C38B—C39B—C40B	57.0 (3)
C57A—O3A—C38A—C37A	−93.3 (2)	C38B—C39B—C40B—C41B	−55.7 (3)
C36A—C37A—C38A—O3A	−175.6 (2)	C39B—C40B—C41B—C54B	−67.6 (2)
C36A—C37A—C38A—C39A	−57.8 (3)	C39B—C40B—C41B—C42B	169.56 (18)
O3A—C38A—C39A—C40A	177.23 (19)	C39B—C40B—C41B—C36B	53.1 (2)
C37A—C38A—C39A—C40A	56.4 (3)	C35B—C36B—C41B—C54B	−63.7 (2)
C38A—C39A—C40A—C41A	−55.5 (3)	C37B—C36B—C41B—C54B	65.8 (2)
C39A—C40A—C41A—C54A	−68.7 (3)	C35B—C36B—C41B—C40B	175.86 (17)
C39A—C40A—C41A—C42A	168.3 (2)	C37B—C36B—C41B—C40B	−54.6 (2)
C39A—C40A—C41A—C36A	52.6 (3)	C35B—C36B—C41B—C42B	57.7 (2)
C35A—C36A—C41A—C54A	−61.7 (2)	C37B—C36B—C41B—C42B	−172.77 (16)
C37A—C36A—C41A—C54A	67.3 (2)	C32B—C33B—C42B—C43B	−52.6 (2)
C35A—C36A—C41A—C42A	59.7 (2)	C34B—C33B—C42B—C43B	−174.56 (17)
C37A—C36A—C41A—C42A	−171.36 (18)	C32B—C33B—C42B—C41B	177.54 (16)
C35A—C36A—C41A—C40A	177.28 (19)	C34B—C33B—C42B—C41B	55.6 (2)
C37A—C36A—C41A—C40A	−53.8 (2)	C54B—C41B—C42B—C43B	−65.0 (2)
C32A—C33A—C42A—C43A	−51.7 (3)	C40B—C41B—C42B—C43B	57.4 (2)
C34A—C33A—C42A—C43A	−174.69 (19)	C36B—C41B—C42B—C43B	173.80 (17)
C32A—C33A—C42A—C41A	179.27 (19)	C54B—C41B—C42B—C33B	62.7 (2)
C34A—C33A—C42A—C41A	56.3 (3)	C40B—C41B—C42B—C33B	−174.92 (16)
C54A—C41A—C42A—C33A	61.2 (2)	C36B—C41B—C42B—C33B	−58.5 (2)
C40A—C41A—C42A—C33A	−176.37 (19)	C33B—C42B—C43B—C44B	53.7 (2)
C36A—C41A—C42A—C33A	−59.9 (2)	C41B—C42B—C43B—C44B	−177.80 (18)
C54A—C41A—C42A—C43A	−67.1 (2)	C42B—C43B—C44B—C45B	−55.8 (3)
C40A—C41A—C42A—C43A	55.4 (3)	C43B—C44B—C45B—C55B	−67.6 (2)
C36A—C41A—C42A—C43A	171.84 (19)	C43B—C44B—C45B—C32B	54.5 (2)
C33A—C42A—C43A—C44A	52.2 (3)	C43B—C44B—C45B—C46B	165.12 (18)
C41A—C42A—C43A—C44A	−179.1 (2)	C31B—C32B—C45B—C44B	170.26 (18)
C42A—C43A—C44A—C45A	−55.4 (3)	C33B—C32B—C45B—C44B	−57.7 (2)
C43A—C44A—C45A—C55A	−65.7 (3)	C31B—C32B—C45B—C55B	−68.8 (2)
C43A—C44A—C45A—C32A	56.5 (3)	C33B—C32B—C45B—C55B	63.2 (2)
C43A—C44A—C45A—C46A	167.5 (2)	C31B—C32B—C45B—C46B	48.26 (19)
C33A—C32A—C45A—C44A	−60.2 (2)	C33B—C32B—C45B—C46B	−179.69 (17)
C31A—C32A—C45A—C44A	168.09 (19)	C44B—C45B—C46B—C47B	82.1 (2)
C33A—C32A—C45A—C55A	60.6 (2)	C55B—C45B—C46B—C47B	−45.0 (3)
C31A—C32A—C45A—C55A	−71.1 (2)	C32B—C45B—C46B—C47B	−163.17 (19)
C33A—C32A—C45A—C46A	177.97 (18)	C44B—C45B—C46B—C30B	−153.63 (18)
C31A—C32A—C45A—C46A	46.3 (2)	C55B—C45B—C46B—C30B	79.3 (2)
C31A—C30A—C46A—C47A	153.2 (2)	C32B—C45B—C46B—C30B	−38.91 (19)
C31A—C30A—C46A—C45A	24.1 (2)	C31B—C30B—C46B—C47B	147.13 (18)
C44A—C45A—C46A—C47A	75.6 (3)	C31B—C30B—C46B—C45B	16.7 (2)
C55A—C45A—C46A—C47A	−51.1 (3)	C45B—C46B—C47B—C56B	−54.6 (3)
C32A—C45A—C46A—C47A	−169.67 (19)	C30B—C46B—C47B—C56B	−175.46 (19)
C44A—C45A—C46A—C30A	−157.0 (2)	C45B—C46B—C47B—C48B	−179.08 (19)
C55A—C45A—C46A—C30A	76.3 (2)	C30B—C46B—C47B—C48B	60.0 (2)
C32A—C45A—C46A—C30A	−42.3 (2)	C56B—C47B—C48B—C49B	84.6 (3)
C30A—C46A—C47A—C56A	163.0 (2)	C46B—C47B—C48B—C49B	−149.1 (2)
C45A—C46A—C47A—C56A	−76.4 (3)	C47B—C48B—C49B—C50B	175.8 (2)
C30A—C46A—C47A—C48A	39.0 (3)	C48B—C49B—C50B—C51B	−178.7 (2)
C45A—C46A—C47A—C48A	159.7 (2)	C49B—C50B—C51B—C53B	65.0 (4)
C56A—C47A—C48A—C49A	65.2 (3)	C49B—C50B—C51B—C52B	−170.6 (3)
C46A—C47A—C48A—C49A	−170.3 (2)	C38B—O3B—C57B—O4B	4.5 (5)
C47A—C48A—C49A—C50A	168.3 (2)	C38B—O3B—C57B—C58B	−174.0 (3)
C48A—C49A—C50A—C51A	175.5 (2)	C9A—O1A—C28A—O2A	0.5 (6)
C49A—C50A—C51A—C52A	−172.5 (2)	C28X—O1A—C28A—O2A	−120 (3)
C49A—C50A—C51A—C53A	64.0 (3)	C9A—O1A—C28A—C29A	179.2 (4)
C38A—O3A—C57A—O4A	3.8 (4)	C28X—O1A—C28A—C29A	59 (3)
C38A—O3A—C57A—C58A	−175.8 (2)	C28Y—O1B—C28B—O2B	−145 (5)
C6B—N1B—N2B—C35B	110.9 (2)	C9B—O1B—C28B—O2B	0.0 (7)
C17B—C1B—C2B—C3B	12.0 (3)	C28Y—O1B—C28B—C29B	35 (5)
C1B—C2B—C3B—C4B	−165.7 (2)	C9B—O1B—C28B—C29B	179.8 (5)
C1B—C2B—C3B—C16B	−36.8 (2)	C28B—O1B—C28Y—O2Y	78 (5)
C2B—C3B—C4B—C5B	−52.8 (3)	C9B—O1B—C28Y—O2Y	−33 (10)
C16B—C3B—C4B—C5B	−177.17 (18)	C28B—O1B—C28Y—C29Y	−70 (9)
C2B—C3B—C4B—C13B	−176.2 (2)	C9B—O1B—C28Y—C29Y	179 (6)
C16B—C3B—C4B—C13B	59.4 (2)	C28A—O1A—C28X—O2X	100 (4)
C3B—C4B—C5B—C6B	−174.69 (17)	C9A—O1A—C28X—O2X	−11 (5)
C13B—C4B—C5B—C6B	−52.7 (2)	C28A—O1A—C28X—C29X	−63 (6)
N2B—N1B—C6B—C7B	178.36 (17)	C9A—O1A—C28X—C29X	−174 (6)
